# Ethnomedicinal uses, phytochemistry and pharmacological aspects of the genus *Premna*: a review

**DOI:** 10.1080/13880209.2017.1323225

**Published:** 2017-05-09

**Authors:** Roza Dianita, Ibrahim Jantan

**Affiliations:** Drug and Herbal Research Center, Faculty of Pharmacy, Universiti Kebangsaan Malaysia, Kuala Lumpur, Malaysia

**Keywords:** Traditional uses, diterpenes, iridoid glycosides, flavonoids, antimicrobial, anti-inflammatory, immunomodulatory, cytotoxic

## Abstract

**Context:** The genus *Premna* (Lamiaceae), distributed throughout tropical and subtropical Asia, Africa, Australia and the Pacific Islands, is used in folk medicine primarily to treat inflammation, immune-related diseases, stomach disorders, wound healing, and skin diseases.

**Objectives:** This review exhaustively gathers available information on ethnopharmacological uses, phytochemistry, and bioactivity studies on more than 20 species of *Premna* and critically analyzes the reports to provide the perspectives and directions for future research for the plants as potential source of drug leads and pharmaceutical agents.

**Methods:** A literature search was performed on *Premna* species based on books of herbal medicine, major scientific databases including Chemical Abstract, Pubmed, SciFinder, Springerlink, Science Direct, Scopus, the Web of Science, Google Scholar, and ethnobotanical databases.

**Results:** More than 250 compounds have been isolated and identified from *Premna* species, comprising of diterpenoids, iridoid glycosides, and flavonoids as the most common secondary metabolites, followed by sesquiterpenes, lignans, phenylethanoids, megastigmanes, glyceroglycolipids, and ceramides. Many *in vitro* and *in vivo* studies have been conducted to evaluate the biological and pharmacological properties of the extracts, and isolated compounds of *Premna* species with antimicrobial, antioxidant, anti-inflammatory, immunomodulatory, antihyperglycaemia, and cytotoxic activities.

**Conclusion:** The bioactive compounds responsible for the bioactivities of most plants have not been well identified as the reported *in vivo* pharmacological studies were mostly carried out on the crude extracts. The isolated bioactive components should also be further subjected to more preclinical studies and elaborate toxicity study before clinical trials can be pursued.

## Introduction

The genus *Premna* was previously classified within the family Verbenaceae (Munir 1984), but has been transferred into the family Lamiaceae, subfamily *Viticodeae* (Harley et al. 2004; Olmstead 2010, 2012). Currently, this genus contains 200 species which are mainly distributed throughout tropical and subtropical Asia, Africa, Australia, and the Pacific Islands (Harley et al. 2004). There are 46 species recognized in the *Flora of China* (Tan & Li 2014) and 14 species occurring in the *Flora Malesiana* area (de Kok 2013). The word ‘Premna’ is derived from the Greek ‘*premnon*’, meaning tree stump, which refers to the short and twisted trunks of *P. serratifolia* L., the first collected species of this genus. Based on the shape and number of calyx lobes, the genus *Premna* has been subdivided into five sections: *Holopremna* Briq. (consisting of two subsections: *Thyrsoideae* and *Corymbiferae*), *Odontopremna* Briq., *Gumira* (Rumph. ex Hassk.) Briq., *Premnos* Briq., and *Holochiloma* Briq. (de Kok 2013).

Morphologically most species in the genus *Premna* are small trees or shrubs and rarely found as lianas (*P. trichostoma* Miq.) and pyroherbs (*P. herbacea* Roxb.). Some species have young twigs with a series of small decussate triangular scales at the base which will fall off once the branch is older. The leaves are usually decussate and hairy. A ridge is often present between the petioles. There are two shapes of calyx types. The first one has four isomorphic lobes, the shape remaining largely intact when the flower develops and when the fruits are formed. The second type has 0–5 lobes, usually heteromorphic. There are also two fruit types: a globose drupe-like fruit consisting of four fleshy mericarps with one seed each, and a clavoid, almost single-seeded, drupe-like and consisting of one fleshy mericarp (de Kok 2013).

Our review of the genus *Premna* is based on ethnomedicinal uses, phytochemical investigations, and pharmacological attributes. This review is comprised of more than 20 species of *Premna* from 150 publications. It is noted that some species have recently been considered as synonyms based on current plant taxonomy (The Plant List 2013). For example: *P. obtusifolia* R.Br., *P. integrifolia* Willd., and *P. corymbosa* var. *obtusifolia* (R.Br.) H.R. Fletcher are synonyms to *P. serratifolia*; *P. japonica* Miq. is a synonym to *P. microphylla* Turcz.; *P. latifolia* Roxb. as a synonym to *P. mollissima* Roth. However, in order to avoid any confusion, we continue to use the species names as referred to by the author(s) of the original papers. The detailed information gathered and critically analyzed in this review should be useful as reference for phytochemists, pharmacologists, medicinal chemists, biochemist, and food scientists to develop the bioactive compounds of the plants as potential nutraceutical, food additives, and pharmaceutical agents.

### Ethnopharmacological uses

The diversity of species of *Premna* throughout the habitat region resulted in various traditional uses by the local people. The earliest report was on ethnomedicinal values of ten species of *Premna* throughout East and Southeast Asia, notably to treat malaria, stomach disorders, headache, cough, malaria and tuberculosis (Perry & Metzger 1980). Most lately, the extensive work by Quattrocchi (2012) has recorded various ethnomedicinal uses of 29 species of *Premna* from numerous regions. Unlike other species which are endemic in certain region, *P. serratifolia* is widely distributed throughout the habitat region which explained its popularity in traditional medicine to treat various diseases or illnesses. In tropical Asia and East Africa, this species is notably used to treat neuralgia and headache, stomachic, fevers, colds and cough, and also to improve liver- and cardiac-related problems (Quattrocchi 2012). Other species, such as *P. tomentosa* Willd., are mostly used to treat stomach-related disorders by local people in Southeast Asia region. The local people in Burma, Thailand, Malay Peninsula and Indonesia use the leaves, root or the inner bark to relieve stomach ache discomfort/pain, for diuretic, or to treat diarrhea (Perry & Metzger 1980; Wiart 2000; Quattrocchi 2012).

Meanwhile, in Polynesian Islands, *P. serratifolia* is commonly used to treat infectious-related diseases such as leuchorrea, genital disease, cancer sores, bad breath and white tongue (Girardi et al. 2015). It is an interesting fact that few species were used in malarial treatment in different regions. For example, bark of *P. angolensis* Gürke was among traditional plants used to treat malaria and other fevers in S. Tomé and Príncipe islands in the Gulf of Guinea (do Céu de Madureira et al. 2002). The bark and the leaves of *P. chrysoclada* (Bojer) Gürke were used in treatment of malaria by the traditional health practitioners in Kilifi District, Kenya (Gathirwa et al. 2011). Quattrocchi (2012) has listed two species of *Premna* that were used in malarial treatment in traditional medicine, *P. foetida* Reinw. Ex Blume leaves used in local communities in topical Asia, and *P. glandulosa* Hand.-Mazz. leaves used by the local community in China.

In the Phillipines, the leaves of *P. odorata* Blanco are used to treat phlegm and tuberculosis (Lirio et al. 2014). In China, India, Vietnam, Burma and Thailand, a few species have been recorded to treat skin diseases such as eczema, ringworms and boils, scabies, skin’s rashes and itching (Perry & Metzger 1980; Quattrocchi 2012; Sharma et al. 2014). The mucillagenous substance of *P. ligustroides* Hemsl. was recorded to be used topically as a sunstrike prophylactic in China (Perry & Metzger 1980). Jeevan Ram et al. (2004) also reported the use of the stem bark of *P. latifolia* for wound healing. Khare (2004, 2007) has highlighted four species of *Premna* (*P. herbacea, P. integrifolia*, *P. latifolia* and *P. tomentosa*) that are used in Ayurvedic medicine, either alone or together with other plant(s), and still available as over-the-counter medicine for local people. Known as ‘agnimantha’, ‘siru thekku’, ‘ghantu bharangin’, ‘agethu’, or ‘gineri’, the decoction of the leaves, stem bark, or roots have been used to treat asthma, rheumatism, neuralgia, diarrhea and stomach disorder, hyperglycaemic, and obesity. It is also used as a post-delivery tonic for women.

The details of species, part of the plant and the ethnomedicinal use of the *Premna* species are detailed in Table 1. Thus, we can categorize the ethnomedicinal values of the *Premna* species (i) as anti-inflammatory – either to treat asthma, rheumatism, gout, pains, fevers; (ii) to improve immune system and treat cold and cough; (iii) for stomach disorders such as diarrhea, dysentery, febrifuge, stomachache; (iv) for wound healing and treating skin diseases; (v) to treat bacterial (for example, tuberculosis, leuchorrea) and malarial infections; (vi) to treat migraine, headache, and neuralgia problems; and (vii) to treat hypertension, diabetes, liver-and cardiac-related problems.

**Table 1.** Some ethnomedicinal uses of *Premna* species

Species	Part of plant	Uses	Community/area	References
*P. angolensis* Gürke	Bark	To treat malaria	S. Tome and Principe islands	do Céu de Madureira et al. 2002
	Leaves	As insect repellent	Africa & Benin	Adjalian et al. 2015
*P. barbata* Wall. ex Schauer	Fruits	Fever, childblain, eczema	India, Pakistan	Quattrocchi 2012
	Wood	Wound healing	India, Pakistan	Quattrocchi 2012
	Stem bark	In throat pain	India, Pakistan	Quattrocchi 2012
*P. bengalensis* C.B.Clarke	Leaves	Improve immune system	India	Quattrocchi 2012
	Bark	In paralysis	India	Quattrocchi 2012
*P. chrysoclada* (Bojer) Gürke	Leaves, roots	Kidney diseases, venereal infections, fevers, dysentry	Tropical Africa	Quattrocchi 2012
	Roots & leaves	To treat malaria; diarrhoea	Kilifi district, Kenya	Gathirwa et al. 2011
*P. cordifolia* Roxb.	Leaves, roots	Febrifuge	Malay peninsula	Perry & Metzger 1980
	Leaves	Anti-inflammatory, rheumatism	Vietnam, Malay peninsula	Quattrocchi 2012
	Roots	Stomachache, diarrhea	Vietnam, Malay peninsula	Quattrocchi 2012
*P. corymbosa* Rottler & Willd.	Leaves	To treat malaria	China	Perry & Metzger 1980
	–	Applied to contusions	Taiwan	Perry & Metzger 1980
	Roots	For stomach disorders	Indo-China	Perry & Metzger 1980
	Leaves	As galactogogue	Indonesia	Perry & Metzger 1980
	–	Cough, headache	Philippines	Perry & Metzger 1980
	–	Headache	New Guinea, Solomon Islands	Perry & Metzger 1980
*P. crassa* Hand.-Mazz.	–	For skin diseases	China, Vietnam	Quattrocchi 2012
*P. cumingiana* Schauer	Leaves	As diuretic, for dropsy and general malaise	Malesia, Philippines	Perry & Metzger 1980; Quattrocchi 2012
*P. divaricata* Wall. ex Schauer	Leaves	For cold	Malay peninsula	Wiart 2000
*P. esculenta* Roxb.	Root	Urinary problem, to espel the stones	India, Thailand	Quattrocchi 2012
*P. foetida* Reinw. ex Blume	Roots	For shortness of breath, cough	Sumatera, Indonesia; Malay peninsula	Perry & Metzger 1980; Wiart 2000
	Leaves	As febrifuge	Malay peninsula	Wiart 2000
	Leaves	Malaria, liver and spleen problems, worms and constipation	Tropical Asia	Quattrocchi 2012
*P. glandulosa* Hand.-Mazz.	Leaves	Malaria, liver and spleen problems, worms and constipation	China	Quattrocchi 2012
*P. henryana* (Hand.-Mazz.) C.Y.Wu	–	For cough and colds	China	Quattrocchi 2012
*P. herbacea* Roxb.	Rhizome	To treat cancer	Thailand	Itharat et al. 2004
	Leaves	Headache	China and Tropical Asia	Quattrocchi 2012
	Leaves & roots	Rheumatic pain, cough, fever, cold	China and Tropical Asia	Quattrocchi 2012
	Roots	Ulcers, rheumatism, gout	China and Tropical Asia	Quattrocchi 2012
	Whole plant	To treat sprain,	China and Tropical Asia	Quattrocchi 2012
	Roots & rhizomes	For dropsy, cough, asthma, fever, rheumatism, cholera	China and Tropical Asia	Quattrocchi 2012
*P. hispida* Benth.	Leaves	Fevers, gastrointestinal disorders, body ache, ear-ache, toothache	Tropical Africa	Quattrocchi 2012
*P. latifolia* Roxb./*P. mollissima* Roth.	Stem bark	For wound healing	Eastern Ghats, India	Jeevan Ram et al. 2004
	Root	As a local application after parturition	Burma	Perry & Metzger 1980
*P. ligustroides* Hemsl.	Mucillaginous substance	Used topically as sunstrike prophylactic	China	Perry & Metzger 1980
	–	For febrifuge	China	Quattrocchi 2012
*P. maxima* T.C.E.Fr.	–	Stomachic, febrifuge	Kenya	Quattrocchi 2012
*P. mollissima* Roth.	Stem, stem bark, bark	Eczema, ring-worms and boils, skin diseases, itches, fever	China, tropical Asia	Quattrocchi 2012
	Leaves	Diuretic, aromatic, dropsy, for a bath to reduce body allergy	China, tropical Asia	Quattrocchi 2012
*P. mucronata* Roxb./*P. mollissima* Roth	Bark	To treat ringworm	Uttarakhand, India	Sharma et al. 2014
	Stem	Eczema, ringworm and boils	India	Quattrocchi 2012
	Leaves	For a bath to reduce body allergy	India	Quattrocchi 2012
*P. nauseosa* Blanco	Leaves	For stomach disorders	Philippines	Perry & Metzger 1980; Quattrocchi 2012
*P. odorata* Blanco	Leaves	To treat tb, phlegm, stomachae, headache, and cough. Also as wound healing, paraciticides, to cure tympanites, beri-beri and heart trouble, to relieve abdominal pain and dysentry	Albay Province, Philippine	Lirio et al. 2014; Perry & Metzger 1980
	Leaves, roots, flowers and fruits	Sudorific, analgesic, pectoral, carminative, headache	Philippines, Taiwan	Quattrocchi 2012
*P. obtusifolia* R.Br./*P. serratifolia* L.	Leaves	Malaria, cough	Manus, Papua New Guinea	Larson et al. 2014
*P. parasitica* Blume	Leaves	As tonic after confinement; for fever	Indonesia; Malay peninsula	Perry & Metzger 1980; Wiart 2000
*P. puberula* Pamp.	Stem bark	Mouth blisters	China	Quattrocchi 2012
*P. pyramidata* Wal. ex Schauer	Shoots	Applied externally on abdomen to treat worms	India	Quattrocchi 2012
*P. quadrifolia* Schumach. & Thonn.	Leaves	As insect repellent	Africa & Benin	Adjalian & others 2015
*P. serratifolia* L.	Leaves	As tonic after childbirth	Malay peninsula	Wiart 2000
*P. serratifolia* L.	Leaves	Migraine	North Bougainville, Papua New Guinea	Larson & others 2014
	Leaves	Cough, constipation	Rotuma, Pacific Islands	McClatchey 1996
	Bark	Hypertension, cardiac insufficiency	Rotuma, Pacific Islands	McClatchey 1996
	Bark	Dysentri, stomachache	Siwai andBuin districts, Bougainville, Papua New Guinea; tropical Asia, east Africa	Waruruai et al. 2011
	Leaves, bark	Headache, malaria	Siwai andBuin districts, Bougainville, Papua New Guinea	Waruruai et al. 2011
	Leaves, twigs	Leucorrhea, genital disease, girl's intimate hygiene, vaginal discharge	Marquesas Islands, Polynesian Islands	Girardi et al. 2015
	Aerial parts	Canker sores, bad breath, thrush, white tongue, oral form of epa, including bewitchment, taboo transgression, medicomagic	Marquesas Islands, Polynesian Islands	Girardi et al. 2015
	Leaves	Diabetes/hypoglycaemic, gout	Marquesas Islands, Polynesian Islands, tropical Asia, East Africa	Quattrocchi 2012
	Leaves	Antiparasitic against tb; to trreat migraine and general pains	New Caledonian	Desrivot et al. 2007
	Whole plants	Rheumatism, neuralgia, headache	Tropical Asia and East Africa	Quattrocchi 2012
	Fruits	Cough	Tropical Asia and East Africa	Quattrocchi 2012
	Leaves	Stomachic, colds, fevers, cough, headache, applied externally for body pain	Tropical Asia and East Africa	Quattrocchi 2012
	Roots	Stomachic, tonic, liver problems, cardiac troubles	Tropical Asia and East Africa	Quattrocchi 2012
*P. steppicola* Hand.-Mazz.	–	Skin diseases	China	Quattrocchi 2012
*P. sunyiensis* C.Pei	–	Astringent, stomachic	China	Quattrocchi 2012
*P. szemaoensis* C.Pei	–	Wound healing, stomachic	China	Quattrocchi 2012
*P. tahitensis* Schauer	Bark	Tonics	Pacific	Quattrocchi 2012
*P. tomentosa* Willd.	Root, leaves	For stomachache, to take care of worms, and as bath after childbirth	Malay peninsula	Perry & Metzger 1980; Wiart 2000
	Inner bark	For diarrhea	Indonesia	Perry & Metzger 1980
	Whole plants	Applied externally on caterpillar stings	Burma, Thailand	Quattrocchi 2012
	Leaves	Diuretic, postpartum remedy, for biliousness and abdominal pains, applied locally on scabies, skin rahses, itching	Burma, Thailand	Quattrocchi 2012
	Oil from root	Stomach disorder	Burma, Thailand	Quattrocchi 2012
*P. urticifolia* Rehder	–	Skin disease	China	Quattrocchi 2012

-: not mentioned.

## Phytochemistry

### Essential oils

The genus *Premna* is not widely known to be rich in essential oil content. Nevertheless, previous studies have reported the contents of essential oils in a range of 0.056–0.102% in some *Premna* species (i.e. *P. angolensis*, 0.056%; *P. barbata* Wall. ex Schauer, 0.08–0.1%; *P. coriacea* C.B. Clarke, 0.08%; *P*. *quadrifolia* Schumach. & Thonn., 0.102%; *P. integrifolia,* not determined; *P. tomentosa,* 0.073%) (Narayan & Muthana 1953; Teai et al. 1998; Chanotiya et al. 2009; Rahman et al. 2011; Sadashiva et al. 2013; Adjalian et al. 2015). Among the compounds identified, 1-octen-3-ol, limonene, α-copaene, β-elemene, β-caryophyllene, and δ-cadinene were found as among well-distributed compounds in studied species in varied concentrations.

### Hydrocarbons, fatty acids, ceramides and glyceoglycolipids

Hydrocarbons and lipid-related constituents [**1–4, 7**] have been identified in *P. fulva* Craib*, P. crassa* Hand.-Mazz.*, P. hainanensis* Chun & F.C.How, *P. odorata*, *P. integrifolia* and *P. serratifolia* (Wei et al. 1991; Hang et al. 2008; Dai et al. 2010; Lirio et al. 2014). A phytochemical study on *P. microphylla* leaves has led to isolation of fatty acids [**5–6**], glyceroglycolipids [**8–10**] and ceramides [**11–12**] (Zhan & Yue 2003). Ceramides and glyceroglycolipid are major components of chloroplast membrane of the plant, which serve mainly as precursors of important signaling compounds/pathways in various cellular processes (Kolter & Sandhoff 1999). A few studies have reported ceramides and glycoglycerolipids to have immunodulatory activity as well as antitumor, anticancer and anti-inflammation properties (Van Veldhoven et al. 1992; Cateni et al. 2004; Ramos et al. 2006; Mbosso et al. 2012).

### Sesquiterpenoids

Habtemariam et al. (1993;) have reported the isolation of an antibacterial sesquiterpenoid, 7α-hydroxy-6,11-cyclofarnes-3(15)-en-2-one [**13**] from *P. oligotricha* Baker. Meanwhile, numerous monocyclofarnesane sesquiterpenes [**14–19, 23–24**] were isolated from *P. microphylla* leaves (Hu et al. 2013). An eudesmane [**25**] and an aromadendrane [**26**] were reported to be isolated from *P. obtusifolia* (Salae et al. 2012). In addition, Sudo et al. (2000) reported the isolation of three megastigmane glycosides [**20–22**] from the leaves of *P. subscandens* Merr.

### Diterpenoids

**Figure 1.**
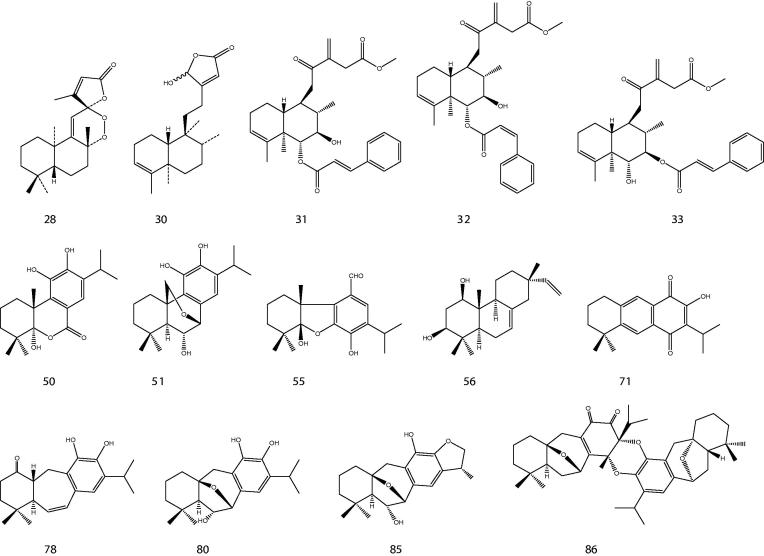
Chemical structures of some diterpenoids obtained from Premna species.

The genus *Premna* is mainly characterized by its diterpenoid constituents (Harley et al. 2004). One study has identified 91 skeletons of diterpenes within Lamiaceae, of which 13 skeletons were frequently identified (Vestri Alvarenga et al. 2001), and abietane diterpenes were highlighted as the most abundant and widespread within Lamiaceae, followed by labdanes, pimaranes, and clerodanes. Interestingly, our current review involving 17 species revealed that icetexanes and abietanes (including *nor*- and *seco*-abietanes) were the most common diterpene types occurred in the genus *Premna*, followed by pimaranes (including iso- and sandaraco-pimaranes), clerodane, labdane, podocarpanes and rosane (Table 2). At one time, icetexanes were found only in three genera of Lamiaceae: *Coleus, Lepechinia*, and *Salvia*. Habtemariam et al. (1990) reported the presence of antibacterial clerodane diterpenes [**29–30**] from the leaves of *P. schimperi* Engl. A year later, two *ent*-labdane diterpenes [**27, 28**] were isolated from the aerial parts of *P. oligotricha* (Habtemariam et al. 1991). Another three clerodanes [**31–33**] were reported in *P. tomentosa* leaves (Chin et al. 2006). The labdane, *ent*-12-oxolabda-8,13(16)-dien-15-oic acid [**27**] and all clerodanes bear a free carboxylic acid unit attached to C-15 with oxygen substitution at C-12 and *sp* ^2^-hybridization between C-13 and C-16. The structures of some of the diterpenes are shown in Figure 1.

**Table 2.** Isolated compounds from genus *Premna* (Lamiaceae).

Classes	No	Isolated compounds	Synonym	Species	References
Fatty acid	**[1]**	Stearic acid	Octadecanoid acid	*P. fulva, P. crassa*	Wei et al. 1990; Wei et al. 1991
Fatty acid	**[2]**	Hexacosoic acid		*P. hainanensis*	Dai et al. 2010
Fatty acid	**[3]**	2-Hexylidene-3-methylsuccinic acid		*P. serratifolia*	Wang et al. 2011
Fatty acid/aldehyde	**[4]**	1-Heneicosyl formate		*P. odorata*	Lirio et al. 2014
Fatty acid	**[5]**	α-Linolenic acid		*P. microphylla*^6^	Zhan & Yue 2003
Fatty acid	**[6]**	1-Monolinolenin		*P. microphylla*^6^	Zhan & Yue 2003
Alkana glucoside	**[7]**	Hexyl glucoside		*P. serratifolia*^2^	Hang et al. 2008
Glyceroglycolipid	**[8]**	1-*O*-(9*Z*,12*Z*,15*Z*-octadecatrienoyl)-3-*O*-β-d-galactopyranosylglycerol		*P. microphylla*^6^	Zhan & Yue 2003
Glyceroglycolipid	**[9]**	Gingerglycolipid A		*P. microphylla*^6^	Zhan & Yue 2003
Glyceroglycolipid	**[10]**	1-*O*-(9*Z*,12*Z*,15*Z*-octadecatrienoyl)-3-*O*-[β-d-galactopyranosyl-(1→6)-*O*-β-d-galactopyranosyl-(1→6)-α-d-galactopyranosyl] glycerol		*P. microphylla*^6^	Zhan & Yue 2003
Ceramide	**[11]**	(2*S*,3*S*,4*R*,11*E*)-2[(2*R*)-2-hydroxytetracosanoylamino]-11-octadecene-1,3,4-triol		*P. microphylla*^6^	Zhan & Yue 2003
Ceramide glucoside	**[12]**	1-*O*-β-d-glucopyranosyl-(2*S*,3*S*,4*R*,8*Z*)-2-[(2*R*)-2-hydroxydocosanoylamino]-8-octadene-1,3,4-triol		*P. microphylla*^6^	Zhan & Yue 2003
Sesquiterpene	**[13]**	7α-Hydroxy-6,11-cyclofarnes-3(15)-en-2-one		*P. oligotricha*	Habtemariam et al. 1993
Sesquiterpene	**[14]**	Blumenol A		*P. microphylla*^6^	Hu et al. 2013
Sesquiterpene	**[15]**	(3*S*,5*R*,6*S*,7*E*,9*R*)-5,6-epoxy-3,9-dihydroxy-7-megastigmene		*P. microphylla*^6^	Hu et al. 2013
Sesquiterpene	**[16]**	3β-Hydroxy-5α,6α-epoxy-7-15-megastigmen-9-one		*P. microphylla*^6^	Hu et al. 2013
Sesquiterpene	**[17]**	Ixerol B		*P. microphylla*^6^	Hu et al. 2013
Sesquiterpene	**[18]**	(−)-Dehydrovomifoliol		*P. microphylla*^6^	Hu et al. 2013
Sesquiterpene	**[19]**	3*S*,5*R*-Dihydroxy-6*S*,7-megastigmadien-9-one		*P. microphylla*^6^	Hu et al. 2013
Sesquiterpene	**[20]**	7-(3,5-Dihydroxy-1,1,5-trimethylcyclohexylidene)-9-methylprop-8-enyl 9-*O*-β-d-glucopyranoside		*P. subscandens*	Sudo et al. 2000
Sesquiterpene	**[21]**	3-Hydroxy-5,6-epoxy-β-ionol 9-*O*-β-d-glucopyranoside		*P. subscandens*	Sudo et al. 2000
Sesquiterpene	**[22]**	2'-*O*-β-d-apiofuranosyl derivative of 3-hydroxy-5,6-epoxy-β-ionol 9-*O*-β-d-glucopyranoside	Premnaionoside	*P. subscandens*	Sudo et al. 2000
Sesquiterpene	**[23]**	Loliotide		*P. microphylla*^6^	Hu et al. 2013
Sesquiterpene	**[24]**	(+)-Dehydrololiolide		*P. microphylla*^6^	Hu et al. 2013
Sesquiterpene	**[25]**	4β,5β-Dihydroxy-10-epi-eudesmane		*P. serratifolia*^3^	Salae et al. 2012
Sesquiterpene	**[26]**	4β,10β-Dihydroxyaromadendrane		*P. serratifolia*^3^	Salae et al. 2012
Diterpene^d^	**[27]**	*ent*-12-Oxolabda-8,13(16)-dien-15-oic acid		*P. oligotricha*	Habtemariam et al. 1991
Diterpene^d^	**[28]**	*ent*-8β,12α-epidioxy-12β-hydroxylabda-9(11),13-dien-15-oic acid γ-lactone		*P. oligotricha*	Habtemariam et al. 1991
Diterpene^c^	**[29]**	(5*R*,8*R*,9*S*,10*R*)-12-oxo-*ent*-3,13(16)-clerodien-15-oic acid		*P. schimperi*	Habtemariam et al. 1990
Diterpene^c^	**[30]**	16α-Hydroxy-cleroda-3,13(14)Z-dien-15,16-diolide			
Diterpene^c^	**[31]**	(5R*,6R*,7R*,8S*,9R*,10R*)-6-*O*-(*trans*-cinnamoyl)-7-hydroxy-12-oxo-3,13(16)-clerodien-15-oic acid methyl ester	Premnone A	*P. tomentosa*	Chin et al. 2006
Diterpene^c^	**[32]**	(5R*,6R*,7R*,8S*,9R*,10R*)-6-*O*-(*cis*-cinnamoyl)-7-hydroxy-12-oxo-3,13(16)-clerodien-15-oic acid methyl ester	Premnone B	*P. tomentosa*	Chin et al. 2006
Diterpene^c^	**[33]**	(5R*,6R*,7R*,8S*,9R*,10R*)-7-*O*-(*trans*-cinnamoyl)-6-hydroxy-12-oxo-3,13(16)-clerodien-15-oic acid methyl ester	Premnone C	*P. tomentosa*	Chin et al. 2006
Diterpene^e^	**[34]**	6α,11,14,16(or 17)-tetrahydroxy-abieta-8,11,13-triene	Nellionol	*P. mollissima*^5^	Rao et al. 1978
Diterpene^e^	**[35]**	6α,11,12,16-tetrahydroxy-7-oxo-abieta-8,11,13-triene		*P. serratifolia*^2^	Yadav et al. 2010
Diterpene^e^	**[36]**	5,6-Double bond of 6α,11,14,16(or 17)-tetrahydroxy-abieta-8,11,13-triene	Anhydronellionol	*P. mollissima*^5^	Rao et al. 1978
Diterpene^e^	**[37]**	5,6-Double bond and enolic 6-OH of 6α,11,14,16(or 17)-tetrahydroxy-abieta-8,11,13-triene	5-dehydronellionol	*P. mollissima*^5^	Rao et al. 1978
Diterpene^e^	**[38]**	6α,11,14,16-tetra-*O*-acetylabieta-8,1,13-trien-7-one	tetracetate of nellionol	*P. mollissima*^5^	Rao & Rao 1981
Diterpene^e^	**[39]**	Abietatrien-1β-ol		*P. serratifolia*^3^	Salae et al. 2012
Diterpene^e^	**[40]**	Abietatrien-1β,12-diol	1β-hydroxy-ferruginol	*P. serratifolia*^3^	Salae et al. 2012
Diterpene^e^	**[41]**	Lambertic acid		*P. serratifolia*^3^	Salae et al. 2012
Diterpene^e^	**[42]**	Ferruginol		*P. serratifolia*^3^	Salae et al. 2012
Diterpene^e^	**[43]**	*O*-Methyl-ferruginol		*P. serratifolia*^3^	Salae et al. 2012
Diterpene^f^	**[44]**	12-Hydroxyabieta-8(14),9(11),12-trien-7-one	Sugiol	*P. serratifolia*^3^	Salae et al. 2012
Diterpene^e^	**[45]**	Royleanone		*P. serratifolia*^3^	Salae et al. 2012
Diterpene^e^	**[46]**	7α,12-Dihydroxy-8,12-abietadiene-11,14-dione	Horminone; 7α-hydroxyroyleanone	*P. serratifolia*^3^	Razak et al. 2010; Salae et al. 2012
Diterpene^e^	**[47]**	Montbretrol		*P. serratifolia*^3^	Salae et al. 2012
Diterpene^e^	**[48]**	5,6,10-Trihydroxy-7-isopropyl-1,1,4α-trimethyl-2,3,4,4α-tetrahydrophenanthren-9(1H)-one	14-deoxycoleone; 6-hydroxy-salvinolone	*P. serratifolia*^3^	Salae et al. 2009; Salae et al. 2012
Diterpene^e^	**[49]**	Taxodion		*P. serratifolia*^3^	Salae et al. 2012
Diterpene^e^	**[50]**	5α,11,12-Trihydroxy-6-oxa-abieta-8,11,13-trien-7-one		*P. serratifolia*^3^	Salae et al. 2012
Diterpene^e^	**[51]**	6α,11,12-Trihydroxy-7β,20-epoxy-8,11,13-abietatriene		*P. serratifolia*^3^	Salae et al. 2012
Diterpene^e*^	**[52]**	Arucadiol		*P. serratifolia*^3^	Salae et al. 2012
Diterpene^e^	**[53]**	11,12,16-Trihydroxy-2-oxo-5-methyl-10-demethyl-abieta-1[10],6,8,11,13-pentene		*P. serratifolia*^1^	Habtemariam and Varghese 2015
Diterpene^f^	**[54]**	12-Hydroxy-6,7-secoabieta-8,11,13-triene-6,7-dial		*P. serratifolia*^3^	Salae et al. 2012
Diterpene^f^	**[55]**	Salvicaranaldehyde		*P. serratifolia*^3^	Salae et al. 2012
Diterpene^g^	**[56]**	13-Formyl-11,14-dihydroxypodocarpa-8,11,13-triene	Premnolal	*P. mollissima*^5^*, P. mollissima*^8^	Rao & Vijayakumar, 1980; Rao et al. 1982
Diterpene^g^	**[57]**	6,7-Dihydropremnolal		*P. mollissima*^8^	Rao et al. 1982
Diterpene^h^	**[58]**	1β,3α,8β-Trihydroxy-pimara-15-ene		*P. serratifolia*^2^	Yadav et al. 2010
Diterpene^h^	**[59]**	2α,19-Dihydroxy-pimara-7,15-diene		*P. serratifolia*^2^	Yadav et al. 2010
Diterpene^h^	**[60]**	Isopimara-7,15-dien-1β,3β-diol		*P. serratifolia*^3^	Salae et al. 2012
Diterpene^h^	**[61]**	Isopimara-7,15-dien-1β,19-diol		*P. serratifolia*^3^	Salae et al. 2012
Diterpene^i^	**[62]**	Sandaracopimar-15-en-8β-ol		*P. mollissima*^5^*, P. mollissima*^8^	Rao & Rao 1978; Rao et al. 1982
Diterpene^i^	**[63]**	Sandaracopimar-15-en-1β,8β-diol		*P. mollissima*^5^*, P. mollissima*^8^	Rao & Rao 1978, Rao & Vijayakumar 1980; Rao et al. 1982
Diterpene^i^	**[64]**	Sandaracopimar-15-en-1β,7α,8β-triol	Previously sandaracopimar-15-en-1β,8β,12β-triol	*P. mollissima*^5^*, P. mollissima*^8^	Rao & Rao 1978; Rao & Vijayakumar 1980; Rao et al. 1982
Diterpene^i^	**[65]**	Sandarocopimar-15-en-7α,8β,11α-triol		*P. mollissima*^8^	Rao et al. 1982
Diterpene^i^	**[66]**	11-*epi*-sandaracopimar-15-en-8β-ol	11-keto-sandaracopimar-15-en-8β-ol	*P. mollissima*^5^	Rao & Rao 1978; Rao & Vijayakumar,^1980^
Diterpene^i^	**[67]**	1-Ketosandaracopimar-15-en-1β,8β-diol		*P. mollissima*^5^	Rao & Vijayakumar 1980
Diterpene^j^	**[68]**	13-*epi*-5,15-rosadien-3α,11β-diol		*P. serratifolia*^3^	Salae et al. 2012
Diterpene^a**^	**[69]**	Obtusinone A		*P. serratifolia*^3^	Salae et al. 2012
Diterpene^a**^	**[70]**	Obtusinone B		*P. serratifolia*^3^	Salae et al. 2012
Diterpene^a**^	**[71]**	Obtusinone C		*P. serratifolia*^3^	Salae et al. 2012
Diterpene^b^	**[72]**	Sirutekkone	Bharangin	*P. herbaceae*	Sandhya et al. 1988; Murthy et al. 2006
Diterpene^a^	**[73]**	8,11,13-Icetexatriene-10,11-12,16-tetrol	Icetexane-1	*P. tomentosa*	Hymavathi et al. 2009
Diterpene^a^	**[74]**	8,11,13-Icetexatriene-10,11,16-triol	Icetexane-2	*P. tomentosa*	Hymavathi et al. 2009
Diterpene^a^	**[75]**	8,11,13-Icetexatriene-7,10,11,16-tetrol	Icetexane-3	*P. tomentosa*	Hymavathi et al. 2009
Diterpene^a^	**[76]**	7,10-Epoxy-8,11,13 icetexatriene-11,12,16-triol	Icetexane-4	*P. tomentosa*	Hymavathi et al. 2009
Diterpene^a^	**[77]**	11,12-Dihydroxy-8,11,13-icetexatrien-1-one		*P. serratifolia*^3^	Salae et al. 2012
Diterpene^a^	**[78]**	11,12-Dihydroxy-6,8,11,13-icetexatatraen-1-one	11,12-dihydroxy-10,6,8,11,13-icetexapentane-1-one	*P. serratifolia*^3^	Razak et al. 2011; Salae et al. 2012
Diterpene^a^	**[79]**	Salviasperanol		*P. serratifolia*^3^	Salae et al. 2012
Diterpene^a^	**[80]**	5,6-Dihydro-6α-hydroxy-salviasperanol		*P. serratifolia*^3^	Asik et al. 2010; Salae et al. 2012
Diterpene^a^	**[81]**	8,11,13-Icetexatriene-10-hydroxy-11,12,16-triacetoxyl	Icetexatriene-1	*P. tomentosa*	Ayinampudi et al. 2012
Diterpene^a^	**[82]**	8,11,13-Icetexatriene-7,10-11-trihydroxy-12,13-dihydofuran	Icetexatriene-2	*P. tomentosa*	Ayinampudi et al. 2012
Diterpene^a^	**[83]**	10β,11-Dihydroxyl-12,16-epoxy-9(10→20)-abeo-abieta-6,8,11,13-tetraene	Latifolionol	*P. mollissima*^5^	Suresh et al. 2011b
Diterpene^a^	**[84]**	10β,11-Dihydroxyl-12,16-epoxy-9(10→20)-abeo-abieta-8,11,13-triene	Dihydrolatifolionol	*P. mollissima*^5^	Suresh et al. 2011b
Diterpene^a^	**[85]**	6β,11-Dihydroxyl-(10→7)β epoxy-12,16-epoxy-9(10→20)-abeo-abieta-8,11,13-triene	Latiferanol	*P. mollissima*^5^	Suresh et al. 2011b
Diterpene^a*^	**[86]**	Obtusinone D		*P. serratifolia*^3^	Salae & Boonnak 2013
Diterpene^a*^	**[87]**	Obtusinone E		*P. serratifolia*^3^	Salae & Boonnak 2013
Diterpene^a*^	**[88]**	Premnalatifolin A		*P. mollissima*^5^	Suresh et al. 2011a
Triterpene	**[89]**	Lupeol		*P. tomentosa; P. hainanensis*	Hymavathi et al. 2009; Ayinampudi et al. 2012; Dai et al. 2010
Triterpene	**[90]**	Betulin		*P. tomentosa*	Hymavathi et al. 2009; Ayinampudi et al. 2012
Triterpene	**[91]**	Lupeol octacosanoate		*P. fulva*	Wei et al. 1991
Triterpene	**[92]**	Lupeol nanocosanoate		*P. fulva*	Wei et al. 1991
Triterpene	**[93]**	Lupeol melissate		*P. fulva*	Wei et al. 1991
Triterpene	**[94]**	Lupene-3one		*P. fulva*	Quan et al. 1989
Triterpene	**[95]**	Friedelin	3-friedelanone	*P. hainanensis, P. fulva, P. crassa*	Quan et al. 1989; Wei et al. 1990, 1991; Dai et al. 2006, 2010
Triterpene	**[96]**	Friedelan-3β-ol	Epifriedelanol	*P. fulva, P. crassa*	Quan et al. 1989; Wei et al. 1990, 1991
Triterpene	**[97]**	Arjunolic acid		*P. microphylla*^6^	Zhan et al. 2009
Triterpene	**[98]**	Hyptatic acid		*P. microphylla*^6^	Zhan et al. 2009
Triterpene	**[99]**	Ursolic acid		*P. tomentosa, P. fulva*	Chin et al. 2006; Dai et al. 2006
Triterpene	**[100]**	Tormentic acid		*P. microphylla*^6^	Hu et al. 2013
Triterpene glycoside	**[101]**	28-*O*-α-l-rhamnopyranosyl (1→2)-β-d-glucopyranoside tormentic acid ester		*P. microphylla*^6^	Zhan et al. 2009
Triterpene glycoside	**[102]**	2α,3β,23-trihdroxy-12,20(30)-ursadien-28-oic acid 28-*O*-β-d-glucopyranosyl(1→2)-β-d-glucopyranosyl ester	Actinicoside	*P. fulva*	Niu et al. 2013
Sterol	**[103]**	β-Ecdysterone		*P. serratifolia*	Wang et al. 2011
Sterol	**[104]**	20,22-Acetonides of inokosterone		*P. serratifolia*	Wang et al. 2011
Sterol	**[105]**	Stigmasterol		*P. odorata, P. mollissima*^5^	Dinda et al. 2010; Lirio et al. 2014
Sterol	**[106]**	β-Sitosterol		*P. odorata; P. mollissima*^5^*; P. fulva; P. hainanensis, P. crassa*	Rao & Rao 1981; Rao et al. 1981; Quan et al. 1989; Wei et al. 1990, 1991; Dai et al. 2006, 2010; Dinda et al. 2010; Lirio et al. 2014
Sterol glycoside	**[107]**	β-Sitosterol-3-*O*-β-d-glucoside		*P. mollissima*^5^	Rao & Rao 1981; Rao et al. 1981; Ghosh et al. 2014;
Sterol glycoside	**[108]**	(3β)-Stigmast-5-en-3-yl β-d-glucopyranoside	β-Daucosterol	*P. hainanensis, P. fulva*	Dai et al. 2006, 2010
Rhamnopyranoside	**[109]**	1-*O-trans-p*-coumaroyl-α-l-rhamnopyranoside		*P. serratifolia*^2^	Hang et al. 2008
Rhamnopyranose	**[110]**	2-*O-trans*-isoferuloylrhamnopyranose		*P. microphylla*^7^	Otsuka et al. 1991c
Rhamnopyranose	**[111]**	3-*O-trans*-isoferuloylrhamnopyranose		*P. microphylla*^7^	Otsuka et al. 1991c
Rhamnopyranose	**[112]**	2-*O-trans-p*-methoxycinnamoylrhamnopyranose		*P. microphylla*^7^	Otsuka et al. 1991c
Rhamnopyranose	**[113]**	3-*O-trans-p*-methoxycinnamoylrhamnopyranose		*P. microphylla*^7^	Otsuka et al. 1991c
Rhamnopyranose	**[114]**	2-*O-cis-p*-methoxycinnamoylrhamnopyranose		*P. microphylla*^7^	Otsuka et al. 1991c
Iridoid glycoside	**[115]**	6-*O*-α-l-(2′′-*O*-caffeoyl) rhamnopyranosylcatalpol		*P. odorata*	Otsuka et al. 1989^a^, 1990b
Iridoid glycoside	**[116]**	6-*O*-α-l-(3′′-*O*-caffeoyl) rhamnopyranosylcatalpol		*P. odorata*	Otsuka et al. 1989^a^, 1990b
Iridoid glycoside	**[117]**	6-*O*-α-l-(2′′-*O*-isoferuloyl) rhamnopyranosylcatalpol		*P. microphylla*^7^	Otsuka et al. 1989c
Iridoid glycoside	**[118]**	6-*O*-α-l-(3′′-*O*-isoferuloyl) rhamnopyranosylcatalpol		*P. microphylla*^7^	Otsuka et al. 1989c
Iridoid glucoside	**[119]**	6-*O*-α-l-(2′′-*O*-caffeoyl) rhamnopyranosylcatalpol		*P. serratifolia*^4^	Otsuka et al., 1993
Iridoid glucoside	**[120]**	6-*O*-α-l-(2′′-*O*-*trans*-*p*-coumaroyl) rhamnopyranosylcatalpol		*P. serratifolia*^4^	Otsuka et al. 1993
Iridoid glucoside	**[121]**	6-*O*-α-l-(2′′-*O*-*cis*-*p*-coumaroyl) rhamnopyranosylcatalpol		*P. serratifolia*^4^	Otsuka et al. 1993
Iridoid glycoside	**[122]**	6-*O*-α-l-(2',3′′-dicaffeoyl) rhamnopyranosylcatalpol	Premnoside A	*P. odorata*	Otsuka et al. 1989b
Iridoid glycoside	**[123]**	6-*O*-α-l-[2-*O*-,3′′-*O*-(or 3′′-*O*-,2′′-*O*-)caffeoyl, *p*-*trans*-coumaroyl] rhamnopyranosylcatalpol	Premnoside B	*P. odorata*	Otsuka et al. 1989b
Iridoid glycoside	**[124]**	6-*O*-α-l-[2-*O*-,3′′-*O*-(or 3′′-*O*-,2′′-*O*-)caffeoyl, feruloyl] rhamnopyranosylcatalpol	Premnoside C	*P. odorata, P. serratifolia*^4^	Otsuka et al. 1989b; Yuasa et al. 1993
Iridoid glycoside	**[125]**	6-*O*-α-l-[2-*O*-,3′′-*O*-(or 3′′-*O*-,2′′-*O*-)feruloyl, *p*-*trans*-coumaroyl] rhamnopyranosylcatalpol	Premnoside D	*P. odorata, P. serratifolia*^4^	Otsuka et al. 1989b; Yuasa et al. 1993
Iridoid glycoside	**[126]**	6-*O*-α-L-(2′′-*O*-isoferuloyl, 4′′-acetyl) rhamnopyranosylcatalpol		*P. microphylla*^7^	Otsuka et al. 1990a
Iridoid glycoside	**[127]**	6-*O*-α-L-(3′′-*O*-isoferuloyl, 4′′-acetyl) rhamnopyranosylcatalpol		*P. microphylla*^7^	Otsuka et al. 1990a
Iridoid glycoside	**[128]**	6-*O*-α-L-(2′′-*O*-*p*-coumaroyl) rhamnopyranosylcatalpol	Saccatoside	*P. microphylla*^7^*; P. serratifolia*^4^	Otsuka et al. 1990b; Yuasa et al. 1993
Iridoid glycoside	**[129]**	6-*O*-α-L-(4′′-*O*-*p*-coumaroyl) rhamnopyranosylcatalpol		*P. microphylla*^7^	Otsuka et al. 1990b
Iridoid glycoside	**[130]**	6-*O*-α-L-(2′′-*O*-*p*-methoxycinnamoyl) rhamnopyranosylcatalpol		*P. microphylla*^7^	Otsuka et al. 1991a,b
Iridoid glycoside	**[131]**	6-*O*-α-L-(3′′-*O*-*p*-methoxycinnamoyl) rhamnopyranosylcatalpol		*P. microphylla*^7^	Otsuka et al. 1991a
Iridoid glycoside	**[132]**	6-*O*-α-L-(2′′-*O*-*p*-methoxycinnamoyl-4′′-*O*-acetyl) rhamnopyranosylcatalpol		*P. microphylla*^7^	Otsuka et al. 1991a
Iridoid glycoside	**[133]**	6-*O*-α-L-(3′′-*O*-*p*-methoxycinnamoyl-4′′-*O*-acetyl) rhamnopyranosylcatalpol		*P. microphylla*^7^	Otsuka et al. 1991a
Iridoid glycoside	**[134]**	6-*O*-α-L-(2′′-*O*-feruloyl) rhamnopyranosylcatalpol		*P. microphylla*^7^	Otsuka et al. 1991b
Iridoid glycoside	**[135]**	6-*O*-α-L-(3′′-*O*-feruloyl) rhamnopyranosylcatalpol		*P. microphylla*^7^	Otsuka et al. 1991b
Iridoid glycoside	**[136]**	6-*O*-α-L-(4′′-*O*-feruloyl) rhamnopyranosylcatalpol		*P. microphylla*^7^*, P. serratifolia*^4^	Otsuka et al. 1991b; Yuasa et al. 1993
Iridoid glycoside	**[137]**	6-*O*-(3′′-*O*-acetyl-2′′-*O*-*trans-p*-coumaroyl)-α-L-rhamnopyranosylcatalpol	Premnacorymbo-side A	*P. serratifolia*^2^	Hang et al. 2008
Iridoid glycoside	**[138]**	6-*O*-(3′′-*O*-*trans*-*p*-coumaroyl)-α-L-rhamnopyranosylcatalpol	Premnacorymbo-side B	*P. serratifolia*^2^	Hang et al. 2008
Iridoid glucoside	**[139]**	1,8-Diester of mussaenosidic acid of 3,7-dimethyloctan-1,8-diol	Premnaodoroside A	*P. odorata, P. serratifolia*^2^	Otsuka et al. 1992; Hang et al. 2008
Iridoid glucoside	**[140]**	3,7-Dimethyloctan-1,8-diol esterified with one moeity each of mussaenosidic acid and 8-*epi*-loganic acid	Premnaodoroside B	*P. odorata*	Otsuka et al. 1992
Iridoid glucoside	**[141]**	3,7-Dimethyloctan-1,8-diol esterified with one moeity each of mussaenosidic acid and gardoside diester	Premnaodoroside C	*P. odorata*	Otsuka et al. 1992
Iridoid glucoside	**[142]**	1,8-Diester of the 8-epiloganic acid of 3,7-dimethyloctan-1,8-diol	Premnaodoroside D	*P. subscandens*	Sudo et al. 1999
Iridoid glucoside	**[143]**	1,8-Diester of the gardoside of 3,7-dimethyloctan-1,8-diol	Premnaodoroside E	*P. subscandens*	Sudo et al. 1999
Iridoid glucoside	**[144]**	Mixture of 1-gardoside-8-epiloganic acid ester of 3,7-dimethyloctan-1,8-diol and 1-epiloganic acid-8-gardoside ester of 3,7-dimethyloctan-1,8-diol (1:1)	Premnaodoroside F	*P. subscandens*	Sudo et al. 1999
Iridoid glucoside	**[145]**	Mixture of 1-gardoside-8-mussaenosidic acid of 3,7-dimethyloctan-1,8-diol and 1-mussaenosidic acid-8-gardoside ester of 3,7-dimethyloctan-1,8-diol	Premnaodoroside G	*P. subscandens*	Sudo et al. 1999
Iridoid glycoside	**[146]**	Bisdesoxydihyromonotropein	7-deoxyloganic acid	*P. mollissima*^5^	Rao et al. 1981
Iridoid glycoside	**[147]**	Geniposidic acid		*P. mollissima*^5^	Rao et al. 1981
Iridoid	**[148]**	Piscrosin D		*P. serratifolia*	Wang et al. 2011
Iridoid glycoside	**[149]**	Aucubin		*P. microphylla*^7^	Otsuka et al. 1991b
Iridoid glycoside	**[150]**	Premnosidic acid		*P. barbata, P. serratifolia*^2^	Negi et al. 2004; Yadav et al. 2013
Iridoid glycoside	**[151]**	10-*O-trans-p*-methoxycinnamoylcatalpol		*P. serratifolia*^2^*, P. subscandens*	Hang et al. 2008; Sudo et al. 1997b
Iridoid glucoside	**[152]**	10-*O-cis-p*-methoxycinnamoylcatalpol		*P. subscandens*	Sudo et al. 1997b
Iridoid glucoside	**[153]**	10-*O-cis-p*-coumaroylcatalpol		*P. subscandens*	Sudo et al. 1997b
Iridoid glucoside	**[154]**	10-*O-trans-p*-coumaroylcatalpol		*P. serratifolia, P. serratifolia*^2^	Wang et al. 2011; Yadav et al. 2013
Iridoid glucoside	**[155]**	10-*O-trans*-caffeoylcatalpol		*P. subscandens*	Sudo et al. 1997b
Iridoid glucoside	**[156]**	10-*O-trans*-isoferuloylcatalpol		*P. subscandens*	Sudo et al. 1997b
Iridoid glucoside	**[157]**	10-*O-trans-p*-methoxycinnamoylasystasioside E		*P. subscandens*	Sudo et al. 1997b
Iridoid glucoside	**[158]**	10-*O-cis-p*-methoxycinnamoylasystasioside E		*P. subscandens*	Sudo et al. 1997b
Iridoid glucoside	**[159]**	10-*O-trans-p*-coumaroylasystasioside E		*P. subscandens*	Sudo et al. 1997b
Iridoid glucoside	**[160]**	10-*O-cis-p*-coumaroylasystasioside E		*P. subscandens*	Sudo et al. 1997b
Iridoid glycoside	**[161]**	10-*O-trans-p*-coumaroyl-6-*O*-α-L-rhamnopyranosylcatalpol		*P. serratifolia*^2^	Yadav et al. 2013
Iridoid glycoside	**[162]**	Scutellarioside II		*P. serratifolia*^2^*; P. subscandens*	Sudo et al. 1997b; Hang et al. 2008
Iridoid glucoside	**[163]**	4′′-Methoxy-*E*-globularinin		*P. subscandens*	Sudo et al. 1998
Iridoid glucoside	**[164]**	4′′-Methoxy-*Z*-globularinin		*P. subscandens*	Sudo et al. 1998
Iridoid glucoside	**[165]**	4′′-Hydroxy-*E*-globularinin		*P. subscandens, P. serratifolia*^2^	Sudo et al. 1998; Yadav et al. 2013
Iridoid glucoside	**[166]**	4′′-Methoxy-*E*-globularimin		*P. subscandens*	Sudo et al. 1998
Iridoid glucoside	**[167]**	4′′-Methoxy-*Z*-globularimin		*P. subscandens*	Sudo et al. 1998
Iridoid	**[168]**	4,4-Dimethoxy-β-truxinic acid catalpol diester		*P. subscandens*	Sudo et al. 2000
Iridoid glycoside	**[169]**	{1-*O*-(3,4-dihydrophenethoxy)-3-*O*-α-L-6-deoxy-mannopyranosyl-4-*O*-[(*E*)-3-(3,4-dihydroxyphenyl)prop-2-enoyl]-β-D-glucopyran-6-yl}oxy-1,4α,5,6,7,7α-hexahydro-6-hydroxy-1-(β-D-glucopyranosyloxy)-7-methylidenecyclopenta[c]pyran-4-carboxylate	Premfulvaoside	*P. fulva*	Niu et al. 2013
Phenethyl alcohol glycoside	**[170]**	Cistanoside F		*P. odorata*	Otsuka et al. 1992
Phenethyl alcohol glycoside	**[171]**	Benzyl alcohol β-D-(2'-*O*-β-D-xylopyranosyl)glucopyranoside		*P. subscandens*	Sudo et al. 2000
Benzyl alcohol glycoside	**[172]**	Phenethyl alcohol β-D-(2'-*O*-β-D-xylopyranosyl)glucopyranoside		*P. subscandens*	Sudo et al. 2000
Phenethyl alcohol glycoside	**[173]**	Acteoside	Verbacoside	*P. serratifolia*^1^*^*,2,4*^, P. microphylla*^7^*, P. odorata, P. subscandens*	Otsuka et al. 1991b, 1992; Otsuka et al. 1993; Yuasa et al. 1993;Sudo et al. 1997a; Hang et al. 2008; Bose et al. 2013
Verbacoside iridoid glucoside	**[174]**	Premcoryoside		*P. serratifolia*^4^*, P. subscandens*	Otsuka et al. 1993; Sudo et al. 1997a
Phenethyl alcohol glycoside	**[175]**	Isoacteoside		*P. odorata*	Otsuka et al. 1992; Yuasa et al. 1993
Phenethyl alcohol glycoside	**[176]**	Martynoside		*P. microphylla*^7^*, P. serratifolia*^4^	Otsuka et al. 1991b; Yuasa et al. 1993
Martynoside glycoside	**[177]**	3-Hydroxy-4-methoxyphenethyl alcohol β-D-(3'-*O*-α-L-rhamnopyranosyl-4'-*O*-β-D-glucopyranosyl-6'-*O*-feruloyl glucopyranoside	Premnafolioside	*P. serratifolia*^4^	Yuasa et al. 1993
Phenethyl alcohol glycoside	**[178]**	Decaffeoylverbascoside	Bioside	*P. odorata, P. subscandens*	Otsuka et al. 1992; Sudo et al. 1997a
Phenylethanoid	**[179]**	Premnethanoside A		*P. subscandens*	Sudo et al. 1997a
Phenylethanoid	**[180]**	Premnethanoside B		*P. subscandens*	Sudo et al. 1997a
Phenolic acid	**[181]**	*p*-Hydroxybenzoic acid		*P. fulva; P. hainanensis*	Chen et al. 2010; Dai et al. 2007, 2010
Phenolic acid	**[182]**	Vanillic acid		*P. fulva*	Wei et al. 1991; Dai et al. 2007; Chen et al. 2010
Aldehyde	**[183]**	4-Hydroxybenzaldehyde		*P. serratifolia*^2^	Hang et al. 2008
Aldehyde	**[184]**	4-Hydroxy-2-methoxybenzaldehyde		*P. serratifolia*^2^	Hang et al. 2008
Aldehyde	**[185]**	Syrangaldehyde		*P. tomentosa*	Hymavathi et al. 2009; Ayinampudi et al. 2012
Aldehyde	**[186]**	Acetoxy syranzaldehyde		*P. tomentosa*	Ayinampudi et al. 2012
Aldehyde	**[187]**	Premnalin		*P. tomentosa*	Ayinampudi 2013
Aldehyde	**[188]**	Coniferaldehyde		*P. tomentosa*	Hymavathi et al. 2009; Ayinampudi et al. 2012
Aldehyde	**[189]**	2-(4-methoxyphenyl)-2-butanone		*P. tomentosa*	Hymavathi et al. 2009; Ayinampudi et al. 2012
Phenolic glucoside	**[190]**	Leonuriside A		*P. serratifolia*^2^	Hang et al. 2008
Alkaloid (indole)	**[191]**	Indole-3-carboxylic acid		*P. microphylla*^6^	Hu et al. 2013
Alkaloid	**[192]**	Premnine		*P. serratifolia*^2^	Basu & Dandiya 1947
Alkaloid	**[193]**	Ganiarine		*P. serratifolia*^2^	Basu & Dandiya 1947
Alkaloid	**[194]**	Aphelandrine		*P. serratifolia*^2^	Dasgupta et al. 1984
Lignan	**[195]**	(+)-Lyoniresinol-2a-*O*-β-D-glucopyranoside		*P. serratifolia*^4^	Yuasa et al. 1993
Lignan	**[196]**	*erythro*-(4-hydroxy-3-methoxyphenyl)-2-{4-[2-formyl-(*E*)-vinyl]-2-methoxyphenoxy}-propan-1,3-diol		*P. serratifolia*^4^	Yuasa et al. 1993
Lignan	**[197]**	*threo*-(4-hydroxy-3-methoxyphenyl)-2-{4-[2-carbinyl-(*E*)-vinyl}-2-methoxyphenoxy)-propana-1,3-diol		*P. serratifolia*^4^	Yuasa et al. 1993
Lignan	**[198]**	Seco-isolariciresinol		*P. recinosa*	Habtemariam et al. 1995
Lignan	**[199]**	Plucheoside D_1_		*P. serratifolia*^4^	Yuasa et al. 1993
Lignan	**[200]**	(+)-Lariciresinol		*P. recinosa*	Habtemariam et al. 1995
Lignan	**[201]**	(−)-Olivil		*P. serratifolia*^4^	Yuasa et al. 1993
Lignan	**[202]**	Premnalatin		*P. mollissima*^5^	Rao & Rao 1981
Lignan	**[203]**	Syringaresinol		*P. fulva*	Dai et al. 2007; Chen et al. 2010
Lignan	**[204]**	(+)-1-Hydroxypinoresinol		*P. recinosa*	Habtemariam et al. 1995
Lignan	**[205]**	(+)-Medioresinol		*P. microphylla*^6^	Hu et al. 2013
Lignan	**[206]**	4-Oxopiresinol		*P. microphylla*^6^	Hu et al. 2013
Lignan	**[207]**	4-*epi*-gummadiol-4-*O*-β-D-glucopyranoside		*P. serratifolia*^4^	Yuasa et al. 1993
Lignan	**[208]**	4β-Hydroxyasarinin-1-*O*-β-glucopyranoside		*P. serratifolia*^2^	Yadav et al. 2013
Lignan	**[209]**	Premnadimer		*P. serratifolia*^2^	Yadav et al. 2013
Xanthone	**[210]**	1-Hydroxy-2,3-methylenedioxy-6-methoxycarbonyl-7-acetylxanthone		*P. microphylla*^6^	Wang & Xu 2003
Xanthone	**[211]**	1,3-Dihydroxy-2-methoxy-6-methoxycarbonyl-7-acetylxanthone		*P. microphylla*^6^	Wang & Xu 2003
Flavonoid	**[212]**	4'-Hydroxy-8,3'-dimethoxy-6-acroleinylflavan-3,4-diol		*P. fulva*	Chen et al. 2010
Flavonoid	**[213]**	Naringenin		*P. fulva, P. recinosa*	Habtemariam et al. 1992; Dai et al. 2007; Chen et al. 2010
Flavonoid	**[214]**	Eriodictyol		*P. recinosa*	Habtemariam et al. 1992
Flavonoid	**[215]**	Pinocembrin		*P. yunnanensis*	Yu et al. 2012
Flavonoid	**[216]**	Pinostrobin		*P. yunnanensis*	Yu et al. 2012
Flavonoid	**[217]**	7-Hydroxy-flavanone		*P. yunnanensis*	Yu et al. 2012
Flavonoid	**[218]**	Apigenin		*P. fulva, P. pyramidata*	Dai et al. 2007; Chen et al. 2010; Monprasart et al. 2011
Flavonoid	**[219]**	5,7-Dihydroxy-4'-methoxy-flavone	Acacetin	*P. odorata, P. szemaoensis*	Li et al. 2008; Pinzon et al. 2011
Flavonoid	**[220]**	Luteolin		*P. serratifolia*^2^*, P. schimperi, P. recinosa*	Habtemariam et al. 1992; Dasgupta et al. 184
Flavonoid	**[221]**	5,7,3'-Trihydroxy-4'-methoxyflavone	Diosmetin	*P. odorata; P. serratifolia*	Pinzon et al. 2011; Wang et al. 2011; Hu et al. 2013; Lirio et al. 2014
Flavonoid	**[222]**	Selagin		*P. pyramidata*	Monprasart et al. 2011
Flavonoid	**[223]**	5-Hydroxy-3’,4’,6,7-tetramethoxyflavone		*P. szemaoensis*	Li et al. 2008
Flavonoid	**[224]**	Quercetin		*P. schimperi, P. recinosa, P. serratifolia*	Habtemariam et al. 1992; Wang et al. 2011
Flavonoid	**[225]**	Kaempferide		*P. schimperi*	Habtemariam et al. 1992
Flavonoid	**[226]**	Myricetin-3’,4’,7-trimethyl ether		*P. tomentosa*	Balakrishna et al. 2003
Flavonoid	**[227]**	3-Methoxy-galangin		*P. yunnanensis*	Yu et al. 2012
Flavonoid	**[228]**	3,7-Dimethoxy-galangin		*P. yunnanensis*	Yu et al. 2012
Flavonoid	**[229]**	5,4’-Dihydroxy-7-methoxyflavonol		*P. szemaoensis*	Li et al. 2008
Flavonoid	**[230]**	3’,4’,5-Trihydroxy-3,7-dimethoxyflavone		*P. szemaoensis*	Li et al. 2008
Flavonoid	**[231]**	5,3’-Dihydroxy-7,4’-dimethoxyflavonol		*P. szemaoensis*	Li et al. 2008
Flavonoid	**[232]**	5,4’-Dihydroxy-3,7,3’-trimethoxyflavone		*P. szemaoensis*	Li et al. 2008
Flavonoid	**[233]**	5-Hydroxy-7,3’,4’-trimethoxyflavonol		*P. szemaoensis*	Li et al. 2008
Flavonoid	**[234]**	Pachypodol	Trimethyl ether of quercetin	*P. recinosa*	Habtemarim et al. 1992
Flavonoid	**[235]**	Chrysosplenol-D		*P. recinosa*	Habtemariam et al. 1992
Flavonoid	**[236]**	3,5,7,5'-Tetrahydroxy-6,3',4'-trimethoxyflavone		*P. oligotricha*	Habtemariam et al. 1992
Flavonoid	**[237]**	3,5,5'-Trihydroxy-6,7,3',4'-tetramethoxyflavone		*P. oligotricha*	Habtemariam et al. 1992
Flavonoid glycoside	**[238]**	Kaempferol-3-*O*-β-D-galactopyranoside		*P. serratifolia*	Wang et al. 2011
Flavonoid glycoside	**[239]**	Quercetin 3-*O*-β-D-xylopyranoside		*P. yunnanensis*	Yu et al. 2012
Flavonoid glycoside	**[240]**	Genkwanin-5-*O*-β-D-glucoside		*P. serratifolia*	Wang et al. 2011
Flavonoid	**[241]**	Vitexin		*P. fulva*	Dai et al. 2007; Chen et al. 2010;
Flavonoid glycoside	**[242]**	Apigenin 7-*O*-β-D-glucopyranoside-4'-acetate		*P. mollissima*^5^	Ghosh et al. 2014
Flavonoid glycoside	**[243]**	Apigenin 7*-O*-β-D-apiofuranosyl (1→2)-α-L-rhamnopyranoside		*P. mollissima*^5^	Ghosh et al. 2014
Flavonoid glycoside	**[244]**	6-*C*-β-D-glucopyranosyl-8-*C*-β-D-xylopyranosyl apigenin	Vicenin-3	*P. tomentosa*	Jyotsna et al. 1984
Flavonoid glycoside	**[245]**	Quercetin 3-rutinoside		*P. serratifolia*^2^	Hang et al. 2008
Flavonoid glycoside	**[246]**	5-Hydroxy-4-methoxy-flavone-7-*O*-bioside		*P. mollissima*^5^	Rao & Rao 1981
Flavonoid glycoside	**[247]**	5-Hydroxy-4'-methoxy-flavone-7-*O*-trioside		*P. mollissima*^5^	Rao & Rao 1981
Flavonoid glycoside	**[248]**	6,3'-Dihydroxy-7-methoxy-4',5'-methylenedioxyisoflavone		*P. microphylla*^6^	Zhong & Wang 2002
Flavonoid glycoside	**[249]**	6,3'-Dihydroxy-7-methoxy-4',5'-methylenedioxyisoflavone-6-*O*-β-D-glucopyranoside		*P. microphylla*^6^	Zhong & Wang 2002
Flavonoid glycoside	**[250]**	6,3'-Dihydroxy-7-methoxy-4',5'-methylenedioxyisoflavone-6-*O*-α-L-rhamnopyranoside		*P. microphylla*^6^	Zhong & Wang 2002
Flavonoid glycoside	**[251]**	6,3'-Dihydroxy-7-methoxy-4',5'-methylenedioxyisoflavone-6-*O*-β-D-xylopyranosyl-(1→6)-β-D-glucopyranoside		*P. microphylla*^6^	Zhong & Wang 2002
Chalcone	**[252]**	2’,4’-Dimethoxy chalcone		*P. yunnanensis*	Yu et al. 2012
Chalcone	**[253]**	Isoliquitirigenin		*P. yunnanensis*	Yu et al. 2012
Chalcone	**[254]**	2-Methoxy isoliquiritigenin		*P. yunnanensis*	Yu et al. 2012
Chalcone	**[255]**	Cardamonin		*P. yunnanensis*	Yu et al. 2012

1 *P. serratifolia* L.

2 *P. serratifolia* L. (syn *P. integrifolia* Willd.).

3 *P. serratifolia* L.(syn *P. obtusifolia* R.Br.).

4 *P. serratifolia* L. (syn *P. corymbosa* var obtusifolia (R.Br.) H.R.Fletcher).

5 *P. mollissima* Roth. (syn *P. latifolia* Roxb.).

6 *P. microphylla* Turcz.

7 *P. microphylla* Turcz (syn *P. japonica* Miq.).

8 *P. mollissima* Turcz (syn *P. latifolia* var. cuneate C.B.Clarke).

a icetexane.

^a^* dimeric icetexane.

^a^** rearranged icetexane.

b quinone methane.

c clerodane.

d labdane.

e abietane.

^e^* nor-abietane.

f secoabietane.

g podocarpane.

h pimarane.

i sandaracopimarane.

j rosane.

Eighteen abietanes [**34–51**], a nor-abietane [**52**], two secoabietanes [**54, 55**] and a abietane [**53**] have successfully been identified in *P. latifolia* (Rao et al. 1978; Rao & Vijayakumar 1980), *P. integrifolia* (Yadav et al. 2010), *P. obtusifolia* (Salae et al. 2012) and *P. serratifolia* (Habtemariam & Varghese 2015). Oxygenated substitution at C-12 of abietane is common within this genus and sometimes the substitution may occur at C-1, C-6, C-7, C-11, C-14 and C-16. While nor-abietane [**52**] is characterized by loss of methyl at C-10, this methyl moves from C-10(α) to C-5(β) in a novel abietane, premnolal [**56**]. Additionally, two abietane derivatives [**56, 57**], known as podocarpanes, were isolated from *P. latifolia* var *cuneata* C.B.Clarke which do not have isoprenyl substitution at C-13. Two pimaranes [**58, 59**] with rare 1,3-dihydroxy and 2-hydroxy, respectively, were isolated from *P. integrifolia* (Yadav et al. 2010). Two isopimaranes [**60–61**] were reported to be identified in *P. obtusifolia* (Salae et al. 2012) along with other pimarane-related type, a rosane [**68]**. Earlier, several studies (Rao & Rao 1978; Rao & Vijayakumar 1980; Rao et al. 1982) reported the occurrence of six sandaracopimaranes [**62–67**] from *P. latifolia* and *P. latifolia* var *cuneata* with common α-hydroxyl substitution at position C-8. The iso- and sandaraco-pimaranes are the isomeric 13-Meβ and 17-Meα forms of pimarane. Though rosane could be found as both 13-C enantiomers, its structure is distinguishable by migration of methyl at C-10 at pimarane to the C-9 position.

Recent studies have identified the genus *Premna* as rich in icetexane diterpenes. As of this review, 20 icetexanes have been isolated from *Premna* species including three dimeric icetexanes and three rearranged icetexanes. Extensive phytochemical work on *P. obtusifolia* has led to the isolation of four icetexanes [**77–80**], two dimeric icetexanes [**86–87**] and three rearranged icetexanes [**69–71**] (Salae et al. 2012; Salae & Boonnak 2013). An icetexane **[72]** was also isolated from *P. herbacea* [**72**] (Sandhya et al. 1988; Murthy et al. 2006), while several icetexanes [**73–76, 81–82**] were obtained from *P. tomentosa* (Hymavathi et al. 2009; Ayinampudi et al. 2012). Icetexanes [**83–85, 88**] were also isolated from *P. latifolia* (Suresh et al. 2011a, 2011b). Hypothetically, icetexane is derived from rearrangement of methyl at C-10 of abietane skeleton to form 6-7-6 tricyclic diterpene. Common substitutions occurs at C-11 and C-12, mostly as hydroxyl [**72–81**] which might further rearrange and form a five-member ring [**82–85**].

### Sterols and triterpenes

Three skeleton type of pentacyclic triterpenes have been reported from the genus *Premna*, i.e. lupane, oleanane and ursane. Three lupane-type diterpenes [**89, 90, 94**] have been identified in *P. fulva* (Quan et al. 1989), *P. hainanensis* (Dai et al. 2010) and *P. tomentosa* (Hymavathy et al. 2009; Ayinampudi et al. 2012) while three derivatives of lupeol [**91–93**] have been isolated from *P. fulva* (Wei et al. 1991). Further studies also reported the presence of four oleanane-type triterpenes [**95–98**] which were distributed in *P. crassa*, *P. fulva*, *P. hainanensis* and *P. microphylla* (Wei et al. 1990, 1991; Dai et al. 2006, 2010; Zhan et al. 2009). Additionally, four ursane-type diterpenes [**99–102**] were identified in *P. fulva* (Dai et al. 2006; Niu et al. 2013), *P. microphylla* (Hu et al. 2013) and *P. tomentosa* (Chin et al. 2006). Common plant sterols, such as stigmasterol [**105**], and their glycosides **[106**,**107**], are widely distributed among *P. crassa, P. fulva*, *P. hainanensis, P. latifolia* and *P. odorata* (Rao et al. 1981; Rao & Rao 1981; Wei et al. 1991; Ghosh et al. 2014; Lirio et al. 2014). Two cholestanes [**103–104]** were isolated from *P. serratifolia* (Wang et al. 2011), and stigmastene-glycoside [**108]** was identified in *P. fulva* (Dai et al. 2006) and *P. hainanensis* (Dai et al. 2010).

### Iridoid and iridoid glycosides

**Figure 2.**
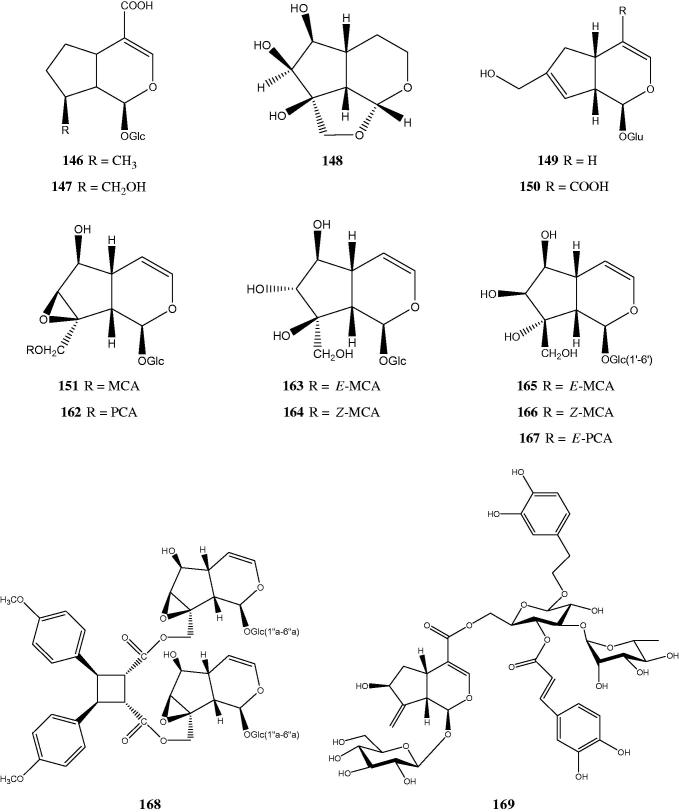
Chemical structures of some of the iridoids and iridoid glycosides.

Iridoids are monoterpene lactones which usually occur in plants as glycosides and sometimes are known as monoterpene alkaloids. They can be found in dicotyledone angiosperms within the superorders Corniflorae, Gentianiflorae, Lamiiflorae and Loasiflorae (Ghisalberti 1998). Their structures are based on cyclopentan[c]pyran skeleton represented as iridane (*cis*-2-oxabicyclo[4.3.0]nonane) and seems to be biosynthesized via alternative cyclization of geranyl diphosphate (Sampaio-Santos & Kaplan 2001). The name ‘iridoid’ itself comes from iridodial and related compounds isolated from the defense secretion of *Iridomyrmex* species (Tietze 1983). Classification of naturally occurring iridoids involves large groups, yet there are four distinguish classes i.e. the non-glycosidic iridoids, iridoid glycosides, iridoid acetal esters, and secoiridoid glycosides. Our current review has identified more than 53 iridoid glycosides within nine species of *Premna* (Table 2). Most of the isolated iridoids are catalpol derivatives [**115–138**, **148–168**] although mussaenosidic acid, epiloganic acid and gardoside derivatives [**139–147, 169**] also could be identified in quite a great number. Majority of the iridoids are linked to their glycosides at C-1 though in catalpol, the glycoside could have linked to C-6. Interesting structure was displayed by compound **168**, with two catalpol glycosides formed an ester to truxinic acid. Piscrosin D [**148**] was the only non-glycoside iridoid isolated from *P. japonica* (Otsuka et al. 1991b) and *P. serratifolia* (Wang et al. 2011), respectively. Figure 2 shows the structures of some of the iridoid and iridoid glycosides.

### Phenylethanoids, aldehydes, alkaloids and lignans

Phenylethanoid glycosides (PhGs) are natural products which are structurally a glycosidic ester consisting of cinnamic acid and hydroxyl phenylethyl moieties attached to glycoside residue. Their structure may consist of monosaccharide, disaccharides, or trisaccharides, with the common glycosides being glucose, rhamnose, xylose, and apiose. They are found in many of the family Lamiaceae where acteoside or verbacoside [**173]** is common (Jiménez & Riguera 1994). Cistanoside F [**170]** and other ten PhGs [**171–180**] were isolated from the genus *Premna* (details in Table 2), of which **174** contains a iridoid moiety attached to its glucose. Phenolic acids [**181, 182**] were reported in *P. fulva* and *P. hainanensis* (Wei et al. 1991; Dai et al. 2007, 2010; Chen et al. 2010) and several aldehydes [**183–190**] were isolated from *P. integrifolia* (Hang et al. 2008) and *P. tomentosa* (Hymavathi et al. 2009; Ayinampudi et al. 2012). One indole carboxylic acid [**191**] was also isolated from *P. microphylla* (Hu et al. 2013). Some alkaloids [**192–194**] were only identified in *P. integrifolia* (Basu & Dandiya 1947; Dasgupta et al. 1984). Lignans, a phenylpropanoid derivatives, were identified within 6 species of *Premna* and commonly found as furan lignans [**199–202]** (Rao & Rao 1981; Yuasa et al. 1993; Habtemariam et al. 1995) and furofuran lignans [**203–209**] (Yuasa et al. 1993; Habtemariam et al. 1995; Dai et al. 2007; Chen et al. 2010; Hu et al. 2013; Yadav et al. 2013) in the genus *Premna* except for compounds **195–198** which are dibenzylbutane lignans (Yuasa et al. 1993; Habtemariam et al. 1995) (Table 2).

### Flavonoids, xanthones and chalcones

**Figure 3.**
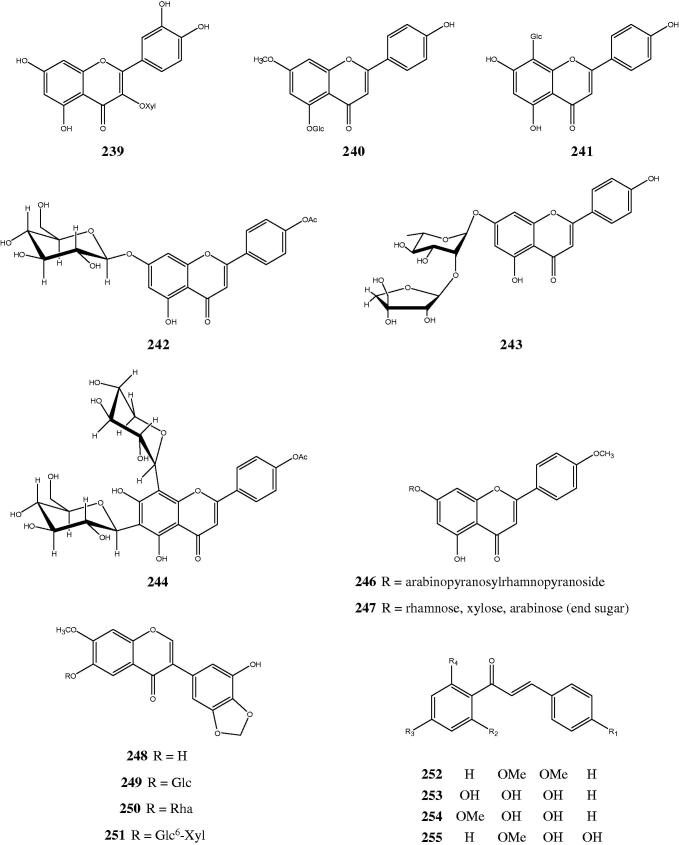
Chemical structures of some flavonoids and flavonoid glycosides found in *Premna* species.

The occurrence of these flavonoids was reported from 13 species (Table 2). Most of the flavonoids were flavonols [**224–239**] and flavones [**218–223, 240–247**], although quite a number were flavanones [**213–217**], isoflavones [**248–251**] and one flavan-3-ol [**212]** (Dasgupta et al. 1984; Habtemariam et al. 1992; Balakrishna et al. 2003; Dai et al. 2007; Li et al. 2008; Chen et al. 2010; Monprasart et al. 2011; Pinzon et al. 2011; Wang et al. 2011; Yu et al. 2012; Hu et al. 2013; Lirio et al. 2014). A few flavonoid glycosides were also reported, identified as *O*-glycoside to either C-3 [**238, 239**], C-5 [**240**], C-6 [**249–251**], or C-7 [**242, 243, 246, 247**]; while two others [**241, 244]** attached to the glycoside residue through C-linkages at C-6 and/or C-8 (Rao & Rao 1981; Jyotsna et al. 1984; Zhong & Wang 2002; Dai et al. 2007; Hang et al. 2008; Chen et al. 2010; Wang et al. 2011; Yu et al. 2012; Ghosh et al. 2014). In addition, two xanthones [**210, 211**] were isolated from *P microphylla* (Wang & Xu 2003) and four chalcones [**252–255**] were reported in *P. yunnanensis* W.W.Sm. (Yu et al. 2012). The structures of some of the flavonoids are shown in Figure 3. The skeleton structure resemblance of the flavonoids (C_6_-C_3_-C_6_), xanthones (C_6_-C_1_-C_6_) and chalcones (C_6_-C_3_-C_6_, without a heterocylic C-ring in the three-carbon α,β-unsaturated carbonyl system) suggested they shared a similar shikimate pathway *via* phenylpropanoid pathway in their biosynthesis whereas xanthones, in particular, might represent the modified shorthened forms of the C_6_-C_3_ system (Dewick 2001; Vogt 2010). However, some references stated that xanthones might possibly derive from shikimate acetate pathways (Velíšek et al. 2008).

## Pharmacological activities

### Antimicrobial, insecticidal, antileishmanial and antimalarial activities

Many studies have been carried out to evaluate the antibacterial and antifungal activities of extracts of *Premna* species (Table 3). Several studies have identified active antimicrobial compounds, mostly found as diterpenes [**27, 29, 30, 48, 51, 55, 69. 70, 72, 78, 79, 80**] (Habtemariam et al. 1990, 1991; Murthy et al. 2006; Salae et al. 2012) and few sesquiterpenes [**13, 25**] (Habtemariam et al. 1993; Salae et al. 2012). Earlier, Kurup and Kurup (1964) has successfully isolated orange crystal substance from the alcoholic extract of the root bark of *P. integrifolia* that was active against *Micrococcus aureus, Bacillus subtillis* and *Streptococcus haemolyticus* (MIC 0-25 μg/mL) but inactive towards *Escherichia coli, Salmonella typhosa* and *B. dysentriae*.

**Table 3.** Antimicrobial and anti-inflammatory effects of the extracts of *Premna* species.

Species (ref.)	Part of plant	Pharmacological effect	Dose, methods & findings
*P. barbata* (Tamta et al. 2012)	Leaves	Antimicrobial	Concentration: 33 mg/200 μL. Methods: Agar disc diffusion Findings: EtOH extract showed weak to moderate activity towards *Argobacterium tumefaciens, Xanthomonas phaseoli, Bacillus subtilis* & *Erwinia chrysanthemi* but not active against *Escherichia coli.* None of the hexane, CHCl_3_ and water extracts showed antibacterial activity towards all microbial.
*P. cordifolia* (Mohd Nazri et al. 2011)	Leaves	Antimicrobial	Concentration: 10 mg/mL Methods: Agar disc diffusion Findings: EtOH extract showed weak zone inhibition (6 cm) against *E. coli, Staphylococcus aureus, Pseudomonas aeruginosa,* and *Streptococcus pyogens* but not active against *Candida albicans.* No activity was displayed by DCM extract.
*P. integrifolia* (Kurup & Kurup 1964)	Root bark	Antimicrobial	Methods: not detailed Findings: Ether fraction of the alcoholic extract was showing antibacterial activity against *Micrococcus aureus, B. subtilis* and *Streptococcus haemolyticus* (IC_50_ 0.3, 0.3 and 0.25 μg/cm^3^, respectively) but not active against *E. coli, Salmonella typhosa,* and *Bacilus dysentriae.*
*P. integrifolia* (Rahman et al. 2011)	Leaves	Antimicrobial	Concentration: 300 μg/disc Methods: Agar disc diffusion and NCCL methods on nutrient broth for MIC Findings: The essential oil and various extracts (MeOH, EtOAc, CHCl_3_ and hexane) showed antibacterial activities towards *Sarcina lutea, B. subtilis, E. coli, Pseudomonas* sp, *Klebsiella pneumonia* and *X. campestries* which were comparable to streptomycin 20 μg/disc. The MIC of the extracts were determined and compared with pure compounds, i.e α-humulene, spathulenol and eugenol.
*P. latifolia* (Jeevan Ram et al. 2004)	Leaves	Antimicrobial	Concentration: 1500 μg/disc Methods: Agar disc diffusion method Findings: EtOH extract exhibited zone inhibition (8-10 mm) against the growth of *P. aeruginosa, S. aureus, M. luteus, M. roseus* and *C. albicans*.
*P. microphylla* (Xu et al. 2010)	Leaves; stem	Antimicrobial	Methods: Agar disc diffusion method Findings: MeOH extract of the leaves showed antibacterial activity against *S. aureus, B. subtilis, S. pyogens, M. kristinae, E. coli, S. typhi and Vibrio mimicus at* MIC 10 mg/mL, but showing no activity towards *P. aeruginosa* and *Shigella dysentriae.* Meanwhile, MeOH extract of the stems only showed antibacterial activity (MIC 10 mg/mL) against *B. subtilis, S. pyogens* and *E. coli.*
*P. serratifolia* (Rajendran & Basha 2010)	Root	Antimicrobial	Concentration: 133 mg/mL Methods: Agar disk diffusion method Findings: Various extracts (hexane, CHCl_3_, EtOAc, EtOH and aqueous) showed antmicrobial activities towards bacteria (*S. aureus,* coagulase negative *Staphylococcus, E. coli, K. pneumonia, P. aeruginosa, S. typhi, S. paratyphi A, S. paratyphi B, V. cholera, Entero cocci*) and fungus (*C. albicans, Aspergillus flavus, Epidermatophyton flocossum, Penicillium chrysogenum, Microsporum gypseum*). The zone inhibition at MIC displayed moderate to high (range 10–25 mm) antibacterial activity towards all tested microorganisms.
*P. serratifolia* (Rajendran 2010)	Bark; wood	Antimicrobial	Concentration: 200 μg/disc Methods: Agar disc diffusion method Findings: Various extracts (hexane, CHCl_3_, EtOAc, EtOH and aqueous) showed moderate to high potency of antimicrobial at against bacteria (*S. aureus,* coagulase negative *Staphylococcus, K. pneumonia, S. typhi, S. paratyphi A, S. paratyphi B, P. aerugiosa, V. cholera*) and fungus *(A. flavus, A. niger, P. notatum, C. albicans*).
*P. corymbosa* (Karthikeyan & Deepa 2011)	EtOH ext.; leaves	Anti-inflammatory	Dose: 200 & 400 mg/kg Methods: egg albumin-induced paw edema (acute inflammation model) and cotton pellet-induced granuloma formation (chronic inflammation model); both in rats. Findings: The extract significantly inhibited the edema in acute inflammation model dose dependently while in chronic model the results indicated mild but significantly decreased granuloma formation (% inhibition 35.17% and 50.38% at doses 200 and 400 mg/kg, respectively).
*P. herbacea* (Narayanan et al. 2000)	EtOH ext.; roots	Anti-inflammatory, Antipyretic, Antinociceptive	Dose: 100, 200, 400 mg/kg Methods: carrageenan-induced paw edema (acute inflammation model) and cotton pellet-induced granuloma formation (chronic inflammation model); both in rats. *Antipyretic*: Typhoid-Paratyphoid A, B (TAB) vaccine-induced pyretic in rabbits. *Antinociceptive*: acetic acid-induced writhing and hot plate tests on mice. Findings: The extract significantly showed antipyretic and antinociceptive effects on particular animal models. The extract did not reduce edema’s volume in the acute inflammation rat and only showing mild yet statistically significant anti-inflammation in chronic model. All, except antinociceptive activity on hot plate test, was shown to be dose dependent.
*P. integrifolia* (Gokani et al. 2011)	MeOH ext.; roots	Anti-inflammatory	Dose: 300 mg/kg Methods*: In vivo*: acute inflammation models (carrageenan-induced edema, histamine-induced wheal formation, formalin-induced edema, acetic acid-induced vascular permeability) and chronic inflammation model (cotton pellet-induced granuloma). *In vitro*: COX-1 inhibitory activity using spontaneous contractions of the rat’s uterus and heat-induced hemolysis of rat’s erythrocytes. Findings: The extract showed significant reduced both acute and chronic edema/granulation in inflammation models which were supported by significant prostaglandin synthase inhibition (% inhibition was 30.43%) on rat’s uterus and stabilization of plasma membrane of rat’s erythrocyte (conc 50, 100 and 150 μg/mL).
*P. integrifolia* (Khatun et al. 2014)	MeOH ext.; barks	Anti-inflammatory and antinociceptive	Dose: 100, 200 mg/kg Methods: carrageenan-induced paw edema, formalin-induced licking response and acetic acid-induced writhing reflex tests. Findings: The extract significantly reduced the writhing reflex and licking response dose dependently. At 200 mg/kg, the extract provided 71.16% inhibition of carrageenan-induced edema.
*P. latifolia* (Mahire et al. 2009)	MeOH ext.; leaves	Anti-inflammatory	Dose: 125, 250 and 500 mg/kg Methods: carrageenan-induced paw edema, cotton pellet-induced granuloma, and acetic acid-induced vascular permeability models. Findings: The extract exhibited significant anti-inflammatory activity on those three animal models, dose dependently.
*P. latifolia* (Kumari et al. 2011)	Water ext.; leaves	Anti-inflammatory	Dose: 9 mL/kg Methods: carrageenan-induced paw edema in rats. Findings: The extract showed significant reduced in the edema after 60 min of the edema induction, and the findings showed better results than *P. obtusifolia* and on par with indomethacin.
*P. obtusifolia* (Kumari et al. 2011)	Water ext.; leaves	Anti-inflammatory	Dose: 9 mL/kg Methods: carrageenan-induced paw edema in rats. Findings: The extract showed significant reduced in the edema after 60 min of edema induction.
*P. obtusifolia* (Salae et al. 2012)	Hexane and CH_2_Cl_2_ ext.; roots	Anti-inflammatory	Concentration: 0, 3, 10, 30 and 100 μg/mL. Methods: LPS-induced nitric oxide (NO) production by murine macrophage-like RAW 264.7 cells. The NO production was measured by using Griess assay. Findings: Both extracts significantly inhibited NO production that comparable to caffeic acid phenylester (positive standard, IC_50_ 5.6 μg/mL), with IC_50_ 4.3 (hexane) and 6.1 (CH_2_Cl_2_) μg/mL.
*P. serratifolia* (Rajendran & Krishnakumar 2010)	EtOH ext.; woods	Antiarthritis	Dose: 300 mg/kg Methods: Freund’s adjuvant-induced arthritis rats, where suspension of killed *Mycobacterium tuberculosis* (0.5%) in liquid paraffin was injected into the left hind paw, and the changes in paw edema were measured. Findings: The extract inhibited the edema by 68.32% after 21 days (indomethacin showed 74.87% inhibition). In hematological parameter, treatment with the extract significantly decreased the total whole blood count (WBC) and erythrocyte & sedimentation rate (ESR), but increased the red blood count (RBC) and hemoglobin (Hb) level.
*P. serratifolia* (Rajagopal et al. 2014)	MeOH ext.; flowers	Anti-inflammatory	Concentration: various, 10-1000 μg/mL Methods: *in vitro* HRBC membrane stabilization, with measured parameter was inhibition of HRBC membrane lysis. Findings: Starting at concentration 100 μg/mL, the extract showed an anti-inflammatory activity with percentage inhibition at 69.41 ± 0.12 μg/mL. The percentage inhibition appeared in linearity with concentration, and at 300 μg/mL, the extract exhibited inhibition at 97.30 ± 0.59 μg/mL.
*P. tomentosa* (Alam et al. 1993)	MeOH ext.; leaves	Anti-inflammatory	Dose: 100 mg/kg Methods: cotton pellet-induced granuloma in rats. Findings: The extract caused a reduction of granuloma by 32.21%, in comparison to phenylbutazone (positive control) which was 33.77%. There was also a decreased in serum protein, SGOT and SGPT.

Other activities such as antioxidant, antidiabetic/antihyperglycaemic, antihyperlipidemic, hepatoprotective and cardioprotective activities are discussed in the main article.

Compound **48** (Salae et al. 2012) appeared to have potent antibacterial activity with most of their MICs were <5 μg/mL, except for *P. aeruginosa*. Interesting broad spectrum antibacterial and antifungal activities were also showed by compound **72** (MIC 5-10 μg/mL), isolated from the roots of *P. herbacea* (Murthy et al. 2006). Another study by Lirio et al. (2014) evaluated antitubercular activity against *Mycobacterium tuberculosis* of the leaves of *P. odorata* and its constituents. Although the extract showed relatively weak inhibitory activity, the fractions exhibited strong activity which eventually led to isolation of the active compound **4** (MIC_90_ 8 μg/mL whilst rifampin 0.05 μg/mL and isoniazid 0.23 μg/mL).

The insecticidal activity of different extracts and essential oil of *P. latifolia* was tested against *Spodoptera litura* larvae, a polyphagus crop pest, by using leaf-dip method. The essential oil showed the highest growth reduction (56.83%) followed by chloroform, hexane and butanol fractions (43.93, 26.01 and 23.69%, respectively) (Kumar et al. 2011). Recent study on *P. angolensis* and *P. quadrifolia* evaluated the insecticidal and repellent effects of its essential oils against *Sitoroga cerealella*, an insect pest of rice stocks, using olfactometer and contact toxicity test (Adjalian et al. 2015). The results showed that both essential oils have insecticidal and repellent activities as indicated by rate of death of *S. cerealella*, percentage of repulsion, number of rice attacked and loss of weight of rice. The leaf extract of *P. serratifolia* showed strong activity against *Leishmania donovani* (IC_50_ 4.4 μg/mL) but showed weak and/or no effect against *Trypanosoma brucei brucei*, *Trichomonas vaginalis* and *Caenorhabditis elegans* (Desrivot et al. 2007). It has been reported previously that clerodane diterpenes [**28** and **29**], isolated from *P. oligotricha* and *P. schimperi*, showed potent antileishmanial effects towards axenically cultured amastigotes of *L. aethiopica* (IC_50_ 1.08 and 4.12 μg/mL, respectively). Both compounds also exhibited high selectivity towards *L. amastigotes* than the permissive host cell line, THP-1 cells or the promastigotes stage of the parasites (Habtemariam 2003).

Although widely used traditionally in malarial treatment by the Philippines, the ethanol extract of *P. angolensis* barks only showed weak antiplasmodial activity (IC_50_ 180–500 μg/mL) towards both chloroquine sensitive and resistant strains of *Plasmodium falciparum* (do Céu de Madureira et al. 2002). However, the leaf extract of *P. chrysoclada* revealed high activity against chloroquinone sensitive and resistant strains of *P. falsiparum* (IC_50_ 7.75 and 9.02 μg/mL) while the root extract only showed moderate activity (IC_50_ 27.63 and 52.35 μg/mL). Further investigation also revealed that the leaf extract (dose 250 mg/mL) has strong ability to reduce the parasitized erythrocyte (9.26% parasitaemia) and to inhibit the parasite growth (65.08% chemo suppression) in *Plasmodium berghei* infected mice (Gathirwa et al. 2011).

### Antioxidant, anti-inflammatory and immunomodulatory activities

*Premna* species are known to have high-antioxidant capacity, such as *P. cordifolia* Roxb. (Mustafa et al. 2010; Mohd Nazri et al. 2011), *P. esculenta* Roxb. (Mahmud et al. 2011), *P. integrifolia* (Gokani et al. 2011; Nguyen & Eun 2011), *P. microphylla* (Xu et al. 2010) and *P. serratifolia* (Rajagopal et al. 2014) (Table 3). The wide distribution of flavonoids and phenolics within this genus seems to contribute to this activity. Various methods were used to measure the antioxidant capacities such as radical scavenging (diphenylpicrylhydrazyl (DPPH), superoxide, nitric oxide NO, hydroxyl radicals), ferric reducing ability of plasma (FRAP), ferric thiocynate (FTC), lipid peroxidation, erythrocyte membrane stabilizing and β-carotene bleaching assays. Most of the radical scavenging capacity of the extracts has been correlated to their phenolic contents – the higher the phenolic content, the higher the antioxidant capacity. The presence of hydroxyl group (OH) and/or unsaturated bond are suggested to play the main role in capturing the radical oxygen species (ROS).

Secondary metabolites such as flavonoids, xanthones, chalcone and other phenolic compounds with high-hydroxyl group substitution are hypothetically contributing to the high antioxidant activity of the plant. For example, two flavone glycosides [**213, 214**] from *P. latifolia* leaves significantly inhibited oxidation of DPPH (IC_50_ 22.5 and 16.0 μg/mL, respectively) (Ghosh et al. 2014). Furofuran lignans [**208**, **209**] and iridoid glycosides [**150, 154, 161, 165**] might contribute to antioxidant activity of the stem bark of *P. integrifolia* when evaluated with radical scavenging (DPPH and NO) and ferric reducing antioxidant power (FRAP) assays (Yadav et al. 2013). Compounds **165** and **154** possessed maximum radical scavenging activity (IC_50_ 0.29 and 0.37 μM) in DPPH assay, followed by compound **209**; while compounds **150** and **161** exhibited maximum reducing power in FRAP assay. Aldehyde derivatives [**186** and **187**] and icetexane diterpenes [**81, 82**] were thought to be potential free radical scavenger constituents from *P. tomentosa* (Ayinampudi et al. 2012; Ayinampudi 2013). The higher number of hydroxyl group in compound **82** (IC_50_ 7.01 μg/mL) than compound **81** (IC_50_ 24.80 μg/mL) reflected the higher antioxidant capacity of the former. Interestingly, this rule was not applied for compound **187** (IC_50_ 20.58 μg/mL) which has three hydroxyl moieties, in comparison to compound **186** (IC_50_ 20.83 μg/mL) which only has one hydroxyl moiety. Potential antioxidant activities were also exhibited by a series of icetexanes [**73-76**] from *P. tomentosa* towards DPPH, NO and superoxide scavenging assays, of which compound **76** demonstrated superior activities than the others and also on par with the standards (Naidu et al. 2014). Recent study also identified an aromatic diterpene [**53]** as antioxidant constituent from *P. serratifolia* with IC_50_ of 20.4 ± 1.3 μM towards DPPH assay (Habtemariam & Varghese 2015).

It is note worthy that although those studies showed some potential antioxidant capacities of some extracts of *Premna* species and its constituents, they do not necessarily reflect the molecular or *in vivo* activities. For example, the DPPH and FRAP assays are mostly based on the simple chemical reaction (Benzie & Strain 1996; Molyneux 2004). These cell-free antioxidant assays do not support the cellular physiological conditions, do not include particular biological substrates that need to be protected, may not encounter the relevant types of antioxidant at molecular level, may not describe the partition coefficient of the compounds, or other cellular factors. Cell-based antioxidant assays are considered more relevant and accurate in representing the *in vivo* conditions since they involve several aspects such as uptake, metabolism, and target site where the compounds might potentially worked within cells (Lü et al. 2010).

Inflammatory reaction occurs due to pathogen invasion into the body or other types of body injury which can cause injury to the tissues or cells as well. At macroscopic level, inflammation is indicated by reddened, swollen, hot, pain, and loss of function of the inflamed area. The loss of function is usually referring to simple loss of mobility in a joint due to pain or edema, or the replacement of functional tissue by the scar tissue. This inflammatory event usually will be followed by the release of mediators from the cells or plasma which modify and regulate the immune response (innate/nonspecific and specific immunological response) (Punchard et al. 2004). Hence, several studies have been conducted to evaluate the anti-inflammatory effect of the extracts of *Premna* species (Table 3). In addition, an extensive study by Salae et al. (2012) identified several compounds from *P. obtusifolia* roots that exhibited potent anti-inflammation activity. Of 20 isolated compounds, four diterpenes [**48, 49, 69, 70**] showed potent *in vitro* lipopolysaccharide (LPS) induced NO inhibitor (IC_50_ 6.1, 7.8, 1.7 and 6.2 μM) that were comparable to positive control, caffeic acid phenylester (IC_50_ 5.6 μM). Meanwhile, megastigmane [**21**] only showed weak anti-inflammatory activity. Further structure-activity relationship analysis suggested that the presence of a hydroxyl group in an *ortho*-naphtoquinone skeleton provided stronger anti-inflammation activity. It was postulated that these active compounds might be responsible for the strong NO inhibitor activity of the hexane and dichloromethane extracts (IC_50_ 4.3 and 6.1 μg/mL, respectively). Another species, *P. integrifolia*, also showed significant *in vivo* anti-inflammatory activity in both acute and chronic inflammation models; further *in vitro* study suggested inhibition of prostaglandin synthase and stabilization of plasma erythrocyte membrane might play role in the *in vivo* activity (Gokani et al. 2011).

Only one calculogenesis-related study has been carried out on *Premna*. The anticalculogenic activity of *P. latifolia* leaves and stems was evaluated *in vitro* by assessing oxalate crystal growth on gel medium in Hane’s tubes *via* single diffusion method over period of 30 days at the concentrations of 20 and 200 mg/mL (Aravindakshan & Bai 1996). The extract effectively reduced the size of oxalate crystal in comparison to negative control and further analysis by using scanning electron microscope showed development of cracks in the crystal interior and rupture tendency. These results concluded chemolysis as an anticalculogenic mechanism of this extract.

Interesting immunostimulant activity was exhibited by *P. pubescens* Blume and *P. tomentosa* leaves. In their *in vitro* studies, Devi et al. (2003a, 2004a) used rat’s splenic lymphocytes and J770 macrophage cell culture which has been induced by using chromium, Cr(IV), to provide immunosuppressant condition. The results showed *P. tomentosa* inhibited the apoptosis of the Cr(IV)-induced cells by preventing the proliferation of the lymphocytes and the macrophages. At the same time, the extract has significantly reduced the ROS level by increasing the levels of the endogenous antioxidant enzymes such as glutathione (GSH), glutathione peroxide (GPx) and superoxide dismutase (SOD) enzymes, and reducing malondialdehyde (MDA) level. Meanwhile, *in vivo* study by Restuati et al. (2014) in the antigen sheep red blood cell (SRBC)-induced immunostimulant rats, suggested that *P. pubescens* stimulated the immune response by increasing the number of leukocytes, immunoglobulin IgG and IgM, and lysozyme. In addition, the methanol extract of *P. integrifolia* roots also produced significant immunomodulatory activity in both specific and nonspecific immune responses following hemagglutinating antibody titer, plaque forming cell assay, delayed-type hypersensitive response, carbon clearance test (phagocytic activity) and *E. coli*-induced abdominal sepsis parameters (Gokani et al. 2007).

### Cytotoxic activities

Traditional use of *P. herbacea* by the Thai to treat cancer has led to the evaluation of the rhizome extract of this species towards several cancer cell lines such as COR-L23, LS-174 T and MCF-7 (Itharat et al. 2004). The results turned out to be negative. However, another study by Dhamija et al. (2013), showed that the root nodules extract had cytotoxic activity on brine shrimp lethality test (BSLT), Ehrlich ascites carcinoma (EAC) cells (trypan blue dye exclusion assay), and MCF-7 cell lines (3-(4,5-dimethylthiazol-2-yl)-2,5-diphenyltetrazolium bromide (MTT) assay). Ethanol extract and ethyl acetate fraction exhibited the most potent cytotoxic effect and further investigation on EAC-inoculated mice and Dalton’s lymphoma ascites (DLA) mice (250 and 500 mg/kg, orally) led to significant elevation of the mean survival rate and reduction of the solid tumor weight and volume. These findings were supported by hematological and antioxidant parameters. The EAC-inoculated mice model was used to evaluate antitumor activity of the ethanol extract of *P. integrifolia*; the findings were found to be comparable to the standard, 5-flurouracil (20 mg/kg) (Sridharan et al. 2011).

About 20 years ago Habtemariam (1995) isolated diterpenes [**27** and **29]** from *P. oligotricha* and *P. schimperi* and suggested they possess cytotoxic property towards several cancer cell lines such as L929, RAW 264.7, HeLa, Sk.N.SH and ECV 304, with IC_50_ values of 1.5–35 μg/mL. Compound **30** was already known to exhibit a cytotoxic effect. Extensive phytochemical works and cytotoxicity assays on *P. tomentosa* (Chin et al. 2006; Hymavathi et al. 2009; Naidu et al. 2014) have led to the identification of several cytotoxic diterpenes [**31-33, 99, 73–76**]. Compounds [**31–33**] showed cytotoxic activity towards several cancer cell lines, Lu1, LNCaP, and MCF-7, but only **32** and **33** were active on *in vivo* hollow fiber assay towards the cell lines (Chin et al. 2006). Diterpenes [**83-85**] from *P. latifolia* exhibited cytotoxic effect towards HT-29 and Hep-G29 cell lines, especially compound **83** and **84** (IC_50_ 0.04 and 0.18 μg/mL, respectively) (Suresh et al. 2011a). Another study has identified a diterpene [**53]** as one of the responsible compounds for cytotoxic property of *P. serratifolia* (Habtemariam & Varghese 2015). A similar study by Biradi and Hullatti (2015) reported the cytotoxic properties of the extract of *P. integrifolia* and its unidentified compounds.

### Antidiabetic/antihyperglycaemic and antihyperlipidemic activities

So far, four species of *Premna* has been studied for their antidiabetic properties. The most common method was using a chemically-induced diabetic animal model. Alloxan-induced hyperglycemic rats have been used to evaluate antidiabetic activity of ethanol extract of *P. integrifolia* at a dose of 250 mg/kg, to confirm the hypolgycaemic activity of this herbal based on the Indian folk medicines (Kar et al. 2003). This activity was further evaluated by Mali (2013) using cafeteria diet induced mice (inbreed) through various parameters (body-mass index, blood glucose, lipid profile, histology valuation) and comparison with a standard drug (simvastatin). The findings indicated significant protective effect of the roots of *P. integrifolia* at doses of 200 and 400 mg/kg. *P. corymbosa* Rottler & Willd., also reduced blood glucose level in both normolgycaemic and alloxan-induced hyperglycemic rats, at doses of 200 and 400 mg/kg (Dash et al. 2005). Similar studies by Ayinampudi et al. (2012) and Ayinampudi (2013) successfully identified two diterpenes [**81, 82],** and two aldehydes **[185, 187]** that were responsible for antihyperglycaemic activity of *P. tomentosa* root by inhibiting enzyme α-glucosidase *in vitro* (IC_50_ values were 22.58, 9.59, 18.41, and 12.11 μg/mL, respectively). One clinical study, based on the Ayurvedic system, evaluated the effectiveness of *P. obtusifolia* roots as an alternative treatment for diabetics (Ghosh et al. 2009). This 9-month study involved 50 patients with a history of obesity. The results showed significant reduction on body-mass index (BMI), atherogenic index and waist-hip ratio after 6 months while the uric acid and mid-triceps skin fold thickness were significantly reduced after 9 months.

The *in vivo* evaluation of antihyperlipidemic activity of herbal extract is normally done by determining the lipid profiles (LDL, HDL, triglycerides, cholesterol) and histology parameters. As mention earlier, *P. tomentosa* leaves extract showed antihyperlipidemic activity towards the animal model by improving lipid profile and reducing lipid metabolizing enzymes (Devi et al. 2004c). Meanwhile, Mali (2013) reported the effect of *P. integrifolia* roots on lipid profile parameters of caffeinated-diet mice. Additionally, the antihyperlipidemic effect of the leaves and roots of *P. esculenta* was evaluated *in vivo* by using Poloxamer 407-induced hyperlipidemis mice and rats (Mahmud et al. 2011). The study was designed for single dose (mice, 500 mg.kg, i.p) and repeated dose (rats, 4 days, 250 mg/kg, p.o), and the results suggested the extract significantly reduced the serum total triglycerides, total cholesterol, LDL and VLDL levels which were comparable to the standard drug, atorvastatin.

### Hepatoprotective and cardioprotective activities

*Premna tomentosa* has been extensively studied for its hepatoprotective activity. Devi et al. (1998, 2004b, [Bibr CIT0028] c, 2005) have evaluated the possible protection mechanisms of the extract of *P. tomentosa* leaves on acetaminophen-induced hepatoxicity in rats, which suggested *via* (i) reducing ROS and generating endogenous antioxidant enzymes in the liver (e.g. glutathione system, superoxide dismutase, catalase); (ii) improving lipid profile and reducing the activities of lipid metabolizing enzymes; (iii) decreasing the acetaminophen-induced membrane damage so that total membrane-bound ATPases would improve and eventually help maintaining active transport and balancing of Na^+^, Ca^2+^ and K^+^ in the liver and serum; and (iv) protecting the liver against mitochondrial damage as the mitochondrium contains enzymes that would catalyze the production of lipid peroxidation products and other toxic metabolites. Additionally, Hari Prasad et al. (2006) postulated the protective mechanism of *P tomentosa* towards dimethylnitrosamine (DMN)-induced hepatic fibrosis was through decreasing the activation of liver stellate cells and accumulation of collagen and other connective tissue proteins. Recently Naidu et al. (2014) reported that the *in vitro* (using HepG2 cells) and *in vivo* (using tBHP-induced hepatic damage mice) hepatoprotective activity of compound **76** increased the viability of hepatic cells and decreased the elevation of serum transferases (SGOT/SGPT) and oxidative damage, including lipid peroxidation. *P. corymbosa* and *P. serratifolia* also showed protective activity on chemically induced (carbontetrachloride (CCl_4_) and paracetamol, respectively) hepatic damage in rats (Karthikeyan & Deepa 2010; Singh et al. 2011).

Two species, *P. mucronata* Roxb. (Patel et al., 2012; Savsani et al., 2014) and *P. serratifolia* (Rajendran & Saleem 2008), are reported to have cardioprotective activity towards a myocardial infarction rat model. The extracts provided protection to the heart via several mechanisms, i.e., (i) decreasing injured cardiac marker enzymes; blood glucose; heart tissue protein; and heart tissue nucleic acids; as well as (ii) maintaining the electrocardiogram (ECG) pattern and hemodynamics changes, increasing myocardial glycogen and restoring antioxidant status. Further investigation has ruled out cardiac stimulant activities of *P. serratifolia* extracts by significantly supporting positive inotropic and negative chronotropic actions similar to that of β-adrenergic effect, decreasing membrane Na^+^K^+^ATPase and Mg^2+^ATPase and increasing Ca^2+^ATPase (Rajendran et al. 2008). There was only one study reporting the gastroprotective activity of *P. serratifolia* leaves on aspirin-induced ulcer rats (Jothi et al. 2010). The evaluation was carried out at doses of 200 and 400 mg/kg by looking at several parameters: lesion index, total- and free-acidity, and percentage of ulceration. The findings suggested that *P. serratifolia* exhibited significant antiulcer and anti-secretory activities in both applied doses.

### Neuropharmacological acitivities

So far, two studies have evaluated the hypnotic and the neuropharmacological effects of *Premna* species on animal models. Devi et al. (2003b) evaluated the effects of the methanol extract of *P. tomentosa* leaves as a central nervous system (CNS) depressant using potentiation of phenobarbitone-induced hypnotic and locomotor activities on rats. At doses of 400 and 500 mg/kg orally, the extract decreased the locomotor activity and moderately increased the sleeping time, that were comparable to CNS depressant, chlorpromazine (10 mg/kg, i.p) yet significantly different to CNS stimulant, ephedrine hydrochloride (10 mg/kg, i.p). A recent study also evaluated the effect of *P. integrifolia* bark on locomotor activity of the rats in the open field and hole-cross tests (Khatun et al. 2014). The findings suggested that *P. integrifolia* significantly affected locomotor activity of the rats at the doses of 250 and 500 mg/kg, orally on both methods, therefore, might act as CNS depressant.

## Discussion

This review summarizes the phytochemical work of more than 19 species (24 species once the synonyms are considered) of *Premna* with more than 250 secondary metabolites have successfully been isolated and identified. It comprises a high number of diterpenes, iridoid glycosides and flavonoids (glycosides and glycones), followed by sesquiterpenes, lignans, phenylethanoids, megastigmanes, glyceroglycolipids and ceramides. Xanthones and alkaloids were rarely identified though a few studies reported their presence in this genus. Meanwhile essential oils were reported in seven species. The distribution of identified secondary metabolites within the genus *Premna* is shown in Table 3.

Although the *Premna* genus is rich in diterpenes and iridoid glycosides, they were not well distributed within the studies species. Diterpenes were abundant in three species such as *P. mollissima*, *P. serratifolia*, and *P. tomentosa* while iridoid glycosides were reported abundantly in *P. serratifolia*, *P. subscandens* and *P. microphylla*. On the contrary, flavonoids seem to be well distributed among 16 reported species despite of their low number in comparison to other groups. Only a few species such as *P. serratifolia, P. microphylla, P. mollissima, P. fulva* and *P. subscandens,* have been extensively studied for their secondary metabolites. Nonethless, a previous review (Taskova et al. 1997) endorsed terpenoids, iridoids, and flavonoids to be used as taxonomic markers in the family Lamiaceae based on their occurrence in 39 species of 25 genera such as *Sideritis, Stachys, Lamium, Phlomis, Ballota, Salvia, Ajuga, Teucrium*. Thus, diterpenoids (icetexane, abietane, labdane, pimarane types), iridoid glycosides (catalpol derivatives), and flavonoids (flavonols and flavones) can be very useful to characterize the taxonomic markers of the genus *Premna* (Taskova et al. 1997) and to provide the secondary metabolite fingerprint of each species through infrared (IR), thin layer chromatography (TLC), high performance liquid chromatography (HPLC), mass spectroscopy (MS), or nuclear magnetic resonance (NMR) analysis.

Some of the biological and pharmacological studies reported on the studied plants have suggested scientific evidence to justify the various plant uses in traditional medicine. However, adequate biological and pharmacological studies on most of the species in the genus *Premna* have not yet been performed because most, especially in *in vivo* studies, were carried out using their crude extracts (Table 4). For example, none of the bioactive molecules have been identified from the active antimalarial *Premna* species. Similarly, some *Premna* species showed potential *in vivo* antihyperlipidemic, cardioprotective, hepatoprotective, gastroprotective, and neuropharmacological activities which require further studies to determine the active compounds and possible mechanisms for a particular activity. While, numerous isolated compounds have been isolated and evaluated for related biological activites, they were limited to *in* v*itro* studies. No toxicological studies that have been carried out, although some species, such as *P. serratifolia*, have been used in Ayurvedic medicine for a long time.

**Table 4.** Summary of pharmacological activities of *Premna* species.

			Type of study	
Pharmacological activities	Species	Part of plant	*in vitro*	*in vivo*
Antimicrobial	*P. barbata*	Extract (leaves)	√	
	*P. cordifolia*	Extract (leaves)	√	
	*P. herbacea*	Isolated compound from roots	√	
	*P. integrifolia*	Extract (leaves, root-barks)	√	
		Essential oil (leaves)	√	
		Isolated compound from root bark	√	
	*P. latifolia*	Extract (leaves)	√	
	*P. microphylla*	Extract (leaves, stems)	√	
	*P. obtusifolia*	Isolated compounds from roots and twigs	√	
	*P. odorata*	Extract (leaves) and isolated compounds	√	
	*P. serratifolia*	Extract (roots, barks, woods)	√	
Antileishmanial	*P. oligotricha*	Isolated compound	√	
	*P. serratifolia*	Extract (leaves)	√	
	*P. schimperi*	Isolated compound	√	
Antimalarial	*P. angolensis*	Extract	√	
	*P. chrysoclada*	Extracts (leaves, roots)	√	
Insecticidal	*P. angolensis*	Essential oils	√	
	*P. latifolia*	Extract and essential oil	√	
	*P. quadrifolia*	Essential oils	√	
Antioxidant	*P. cordifolia*	Extract	√	
	*P. esculenta*	Extract	√	
	*P. integrifolia*	Extract	√	
		Isolated compounds from stem barks	√	
	*P. latifolia*	Isolated compounds from leaves	√	
	*P. microphylla*	Extract	√	
	*P. serratifolia*	Extract and isolated compounds	√	
	*P. tomentosa*	Isolated compounds	√	
Anti-inflammatory (including antinociceptive and antipyretic)	*P. corymbosa*	Extract (leaves)		√
	*P. herbacea*	Extract (roots)		√
	*P. integrifolia*	Extract (roots)	√	√
		Extract (barks)		√
	*P. latifolia*	Extract (leaves)		√
	*P. obtusifolia*	Extract (leaves)		√
		Extract (roots) and isolated compounds	√	
	*P. serratifolia*	Extract (flowers)	√	
	*P. tomentosa*	Extract (leaves)		√
Anticalculogenic	*P. latifolia*	Extracts (leaves, stems)	√	
Antiarthritis	*P. serratifolia*	Extract (woods)		√
Immunomodulatory	*P. integrifolia*	Extract (roots)	√	√
	*P. pubescens*	Extract (leaves)		√
	*P. tomentosa*	Extract (leaves)	√	√
Cytotoxic activity	*P. herbacea*	Extract (rhizome)	√	
		Extract (root nodule)	√	√
	*P. integrifolia*	Extract		√
		Isolated compound	√	
	*P. oligotricha*	Isolated compounds	√	
	*P. schimperi*	Isolated compounds	√	
	*P. serratifolia*	Isolated compound	√	
	*P. tomentosa*	Isolated compounds	√	
Antidiabetic	*P. corymbosa*	Extract		√
	*P. integrifolia*	Extract (roots)		√
	*P. obtusifolia*	Roots	- Clinical trials -	
	*P. tomentosa*	Isolated compounds	√	
Antihyperlipidemic	*P. esculenta*	Extract (leaves, roots)		√
	*P. integrifolia*	Extract (roots)		√
	*P. tomentosa*	Extract (leaves)		√
Hepatoprotective effect	*P. corymbosa*	Extract		√
	*P. serratifolia*	Extract		√
	*P. tomentosa*	Extract (leaves)		√
		Isolated compound	√	√
Cardioprotective effect	*P. mucronata*	Extract		√
	*P. serratifolia*	Extract		√
Gastroprotective effect	*P. serratifolia*	Extract (leaves)		√
Neuropharmacological activity	*P. integrifolia*	Extract (barks)		√
	*P. tomentosa*	Extract (leaves)		√

There was no effort to qualitatively and quantitatively analyze the extracts used. Standardization of the extracts should be carried out to ensure consistency of the quantitative amounts of the active chemical markers in the plants of similar species collected from different locations. The variety and distribution of active secondary metabolites from this genus are useful as bioactive chemical markers for standardization and quality control purposes. Otherwise the work on biological activities may not be reproducible due to variations in the quantitative amounts of chemical constituents in the plants. These quantitative and qualitative differences in the chemical composition are related to responses of the plants to environmental factors or genetic adaptation of the populations growing at different altitudes to a specific environment (World Health Organization [WHO] 2000, 2003).

## Conclusions and future prospects

Further investigations are required to transform the experience-based claims on the traditional uses of *Premna* species into evidence-based information. The present knowledge obtained mainly from experimental studies was critically assessed to provide evidence and justification for their traditional uses to propose future research prospects for this plant. Phytochemical studies on *Premna* species have led to characterization of diterpenoids, iridoid glycosides, and flavonoids as the charactetistic chemical composition of the genus. The *in vitro* and *in vivo* evaluation of biological properties of the extracts and isolates from various species of *Premna* on antimicrobial, antioxidant, anti-inflammatory, immunomodulatory, cytotoxic, antihyperglycaemic, and other activities should lead to further detailed investigations to identify the bioactive compounds and their mechanisms of action. The antimalarial, hepatoprotective, cardioprotective and gastroprotective effects of the plant extracts should encourage further studies on these plants for use as preventive agents. Toxicological evaluation should be conducted to address any adverse side effects which may occur. The roles and mechanisms of the bioactive compounds should be addressed appropriately to understand the contribution of individual compound to the activities as well as to become potential lead molecules for development into drug candidates. Attempts should be made to carry out more preclinical studies of the standardized extracts and bioactive compounds of *Premna* species, which include determination of modes or mechanisms of action in different animal models, bioavailability, pharmacokinetics and toxicological studies before submission of potential candidates to serious randomized human trials is possible. As more scientific evidences on therapeutic effects are discovered, *Premna* species will be recognized as a valuable source of drug leads and pharmaceuticals.
